# The rare DRB1*04:08-DQ8 haplotype is the main HLA class II genetic driver and discriminative factor of Early-onset Type 1 diabetes in the Portuguese population

**DOI:** 10.3389/fimmu.2023.1299609

**Published:** 2024-01-03

**Authors:** Iris Caramalho, Paula Matoso, Dário Ligeiro, Tiago Paixão, Daniel Sobral, Ana Laura Fitas, Catarina Limbert, Jocelyne Demengeot, Carlos Penha-Gonçalves

**Affiliations:** ^1^ Instituto Gulbenkian de Ciência, Oeiras, Portugal; ^2^ Faculdade de Ciências, Universidade de Lisboa, Lisboa, Portugal; ^3^ Centro de Sangue e Transplantação de Lisboa, Instituto Português do Sangue e Transplantação, Unidade de Imunocirurgia e Imunoterapia, Fundação Champalimaud, Lisboa, Portugal; ^4^ Pediatric Endocrinology Unit, Hospital de Dona Estefânia, Centro Hospitalar Universitário de Lisboa Central (CHULC)/Nova Medical School, Lisbon, Portugal; ^5^ Comprehensive Health Research Centre (CHRC), NOVA Medical School, Universidade Nova de Lisboa, Lisboa, Portugal

**Keywords:** Type 1 diabetes, age of onset, T1D endotypes, Early-onset Type 1 diabetes, HLA class II

## Abstract

**Introduction:**

Early-onset Type 1 diabetes (EOT1D) is considered a disease subtype with distinctive immunological and clinical features. While both Human Leukocyte Antigen (HLA) and non-HLA variants contribute to age at T1D diagnosis, detailed analyses of EOT1D-specific genetic determinants are still lacking. This study scrutinized the involvement of the HLA class II locus in EOT1D genetic control.

**Methods:**

We conducted genetic association and regularized logistic regression analyses to evaluate genotypic, haplotypic and allelic variants in DRB1, DQA1 and DQB1 genes in children with EOT1D (diagnosed at ≤5 years of age; n=97), individuals with later-onset disease (LaOT1D; diagnosed 8-30 years of age; n=96) and nondiabetic control subjects (n=169), in the Portuguese population.

**Results:**

Allelic association analysis of EOT1D and LaOT1D unrelated patients in comparison with controls, revealed that the rare DRB1*04:08 allele is a distinctive EOT1D susceptibility factor (corrected p-value=7.0x10^-7^). Conversely, the classical T1D risk allele DRB1*04:05 was absent in EOT1D children while was associated with LaOT1D (corrected p-value=1.4x10^-2^). In corroboration, HLA class II haplotype analysis showed that the rare DRB1*04:08-DQ8 haplotype is specifically associated with EOT1D (corrected p-value=1.4x10^-5^) and represents the major HLA class II genetic driver and discriminative factor in the development of early onset disease.

**Discussion:**

This study uncovered that EOT1D holds a distinctive spectrum of HLA class II susceptibility *loci*, which includes risk factors overlapping with LaOT1D and discriminative genetic configurations. These findings warrant replication studies in larger multicentric settings encompassing other ethnicities and may impact target screening strategies and follow-up of young children with high T1D genetic risk as well as personalized therapeutic approaches.

## 1 Introduction

Type 1 Diabetes (T1D) is a multifactorial disease with a strong genetic component that results from the immune-mediated destruction of insulin-producing pancreatic beta cells, leading to lifelong insulin dependency. T1D predominantly manifests in childhood and typically presents with a peak of incidence near or at adolescence (10 to 14 years of age) (1, 2).

Numerous studies conducted in diverse populations contributed to uncover the genetic basis of T1D susceptibility. Familial aggregation and genome-wide association studies established that the genomic region located on chromosome 6p21 harboring the Human Leukocyte Antigen (HLA) class II genes, mainly *DRB1*, *DQA1* and *DQB1* accounts for about half of the T1D genetic risk (3, 4). Within Caucasian populations, two HLA haplotypic configurations consistently emerged as the strongest genetic risk factors, namely DRB1*03:01-DQA1*05:01-DQB1*02:01 (referred to as DR3-DQ2) and DRB1*04-DQA1*03-DQB1*03:02 (DR4-DQ8) (4–6). It is also recognized that T1D susceptibility is differentially influenced by the DRB1*04 alleles present within the DR4-DQ8 haplotype. Among these, the DRB1*04:05 allele is associated with the highest risk, followed by DRB1*04:01, DRB1*04:02 and DRB1*04:04, while DRB1*04:03 shows protective effects (6).

T1D susceptibility conferred by distinct HLA alleles has been proposed to result from variation in amino acid residues at specific positions within the peptide-binding groove, which may affect the set of antigenic peptides presented to T cells. Hence, the presence of non-aspartate residues at position 57 of DQB1 and arginine at position 52 of DQA1 was found to confer strong susceptibility to T1D (7, 8). More recently, it has been established that two positions in DRB1 (13 and 71) together with position 57 of DQB1 capture more than 90% of the phenotypic variance attributed to the HLA locus (9), implicating the P4 and P9 pockets in the antigen-binding groove of DRB1 and DQB1, respectively, in T1D risk. In addition to HLA, more than 57 loci located outside of this region were found to contribute to T1D risk with relatively modest effects (10). Interestingly, several of these non-HLA loci have been suggested to influence immune cells or pancreatic beta-cell functions (11).

Recent observations support the notion that T1D is a heterogeneous clinical entity composed of distinct disease subtypes that are distinguished by different pancreatic immunophenotypes and increased clinical severity in patients with younger age at diagnosis (12–15). Accordingly, children diagnosed with T1D under the age of 7 usually present with a more aggressive form of insulitis, characterized by both T and B cell infiltrates whereas children diagnosed at 13 years of age or later, tend to show milder insulitis with predominant T cell infiltration (12). Moreover, children diagnosed under the age of 5 years exhibit reduced residual beta-cell mass at diagnosis and sharply decreased insulin production shortly after disease onset, which is associated with severe metabolic decompensation (13–15). This early-onset disease subtype poses a significant clinical challenge, as these patients are at a higher risk of long-term diabetic complications compared to those with disease onset at older ages (16).

While the genetic landscape of T1D susceptibility has been extensively studied, the search for genetic determinants of age at T1D diagnosis has received comparatively less attention. Nonetheless, disease concordance studies in twins and siblings provide evidence that the age at which individuals develop T1D is strongly influenced by genetic factors (17, 18). These and other reports have contributed to establish that the genetic component impacting on age at T1D diagnosis encompasses both non-HLA and HLA genes (15, 19–26). Non-HLA gene associations include *IL2*, *RNLS*, *PTPRK*, *THEMIS*, *GLIS3*, *IL2RA*, *IL10*, *IKZF3* and *CTSH* (19–22). Notably, individuals diagnosed at a younger age are more likely to carry high-risk HLA class II genetic configurations, including the DR3/DR4 genotype, than those diagnosed at an older age (15, 21–26). Notwithstanding, a detailed comparison of HLA class II gene variants in early-onset versus later-onset disease has been overlooked.

In search for HLA variants that discriminate susceptibility to early-onset T1D (EOT1D), we analyzed HLA class II polymorphisms in a collection of Portuguese children with diagnosis at ≤5 years of age in comparison with individuals diagnosed between 8 and 30 years of age (Later-onset T1D; LaOT1D). Our results revealed that the genetic susceptibility to EOT1D conferred by the HLA class II locus comprised risk factors that overlap with LaOT1D susceptibility and private genetic configurations that mainly pertain to *DRB1* gene variants. Molecular and mechanistic studies are warranted to uncover the role of these genetic factors on age at T1D onset, and may provide novel tools to improve risk prediction, earlier diagnosis, and targeted preventive interventions in individuals at higher risk of developing EOT1D.

## 2 Material and methods

### 2.1 Ethics

Ethical permissions for this study were obtained from the Ethics Committee from Hospital de Dona Estefânia (HDE; Comissão de Ética para a Saúde, Centro Hospital de Lisboa Central, #318/2016), and the Ethics Committee from Associação Protetora dos Diabéticos de Portugal (APDP). All procedures in this study were in accordance with National and European regulations, including the Helsinki Declaration.

### 2.2 Subjects

Subjects with Type 1 diabetes (T1D) with age of disease onset ≤5 years (n=97) were outpatients of the Unidade de Endocrinologia Pediátrica at HDE, that were enrolled between April 2016 and July 2018, and embodied the Early-onset (EO)T1D cohort (80.4% of these diagnosed before 5 years of age). The later-onset cohort (LaOT1D) comprised 96 subjects with ages at T1D onset from 8 to 30 years (83% of those diagnosed before 21 years of age) and were collected at HDE and APDP. Ninety six percent of T1D patients were of European ancestry. T1D diagnosis met the criteria established by the American Diabetes Association (27). All patients were insulin-dependent since diagnosis and had been on uninterrupted insulin treatment. Control subjects with no history of T1D or hyperglycemia (n=169; average age at recruitment 42 years; IQR [35-51] years), and deemed unlikely to develop T1D post-enrolment, were selected within a cohort representative of the Portuguese population [Prevadiab 2 (28)].

### 2.3 Genotyping

DNA extraction was performed from peripheral blood using standard techniques. The HLA-DRB1 and DQA1/DQB1 typing for patients and controls was assessed with a Luminex-based SSOP typing array and Sequence-Based Typing (Sanger) for allelic resolution, according to manufacturer´s protocols (One Lambda LabType SSO and HLAssure, respectively). Haplotypes were reconstructed from allele data using Arlequin v3.5 software (29). Individual extended genotypes carried were derived from reconstructed haplotypes. DR3-DQ2 and DR4-DQ8 were used to classify subjects with the DRB1*03:01-DQA1*05:01-DQB1*02:01 and DRB1*04:01/04:02/04:04/04:05/04:08-DQA1*03:01/03:02-DQB1*03:02 haplotypes, respectively.

### 2.4 Statistical analysis

Allelic, haplotypic and genotypic association tests as well as Odds-Ratio calculations were performed with Plink software package (v1.07), using the BCGene user interface. In this analysis, 8 genotypes; 19 DRB1, 9 DQA1 and 12 DQB1 alleles; and 18 haplotypes were independently evaluated. P-values were computed using the Fisher’s exact test and multi-comparison analyses were corrected with the Holm-Bonferroni method.

To estimate the predictive power of HLA class II haplotypes, regularized logistic regression was performed using scikit-learn python package v1.1 (30). This modeling approach was executed in a cross-validated fashion, where the dataset was partitioned into five blocks. In this process, 80% of the data, corresponding to four blocks, was used for training the model, and the remaining 20%, or one block, was reserved for evaluating model performance. The robustness of the fitting was assessed by reiteration of this process.

## 3 Results

### 3.1 Contribution of HLA class II genotype classes to disease susceptibility in EOT1D and LaOT1D

We used a two-way case-control study design to analyze HLA class II locus variants in 97 unrelated Portuguese subjects with Early-onset T1D (EOT1D, age at diagnosis 0-5 years) and 96 unrelated Portuguese subjects with disease onset after 7 years of age (LaOT1D, age at diagnosis 8-30 years), using as controls 169 non-diabetic individuals, representative of the Portuguese population (**Table 1**). 

**Table 1:** Demographic and clinical characteristics of patients and non-diabetic controls.

*Dataset*	n	Sex (m/f; m%)	Age of onset (mean±std; [IQR])
Early-onset (EO)T1D	97	54/43; 55.7%	2.7±1.5; [1-4]
Later-onset (LaO)T1D	96	56/39; 58.3%	15.3±5.4; [11-18]
Controls	169	82/87; 48.5%	NA

n, number of individuals; m, male; f, female; NA, not applicable.

The clinical manifestation of T1D in preschool children is characterized as more severe when compared to those who develop the disease later in childhood or in adulthood (15, 31, 32). Accordingly, we found evidence that EOT1D patients had lower fasting c-peptide at diagnosis (**Supplementary Table 1**) denoting diminished insulin secretion capacity, presumably due to extensive pancreatic beta-cell autoimmune destruction. Moreover, despite presenting with a lower percentage of glycated hemoglobin at diagnosis, EOT1D patients showed worse metabolic control under insulin treatment, as indicated by the lower proportion of individuals with glycated hemoglobin below 7.5% one year after diagnosis (EOT1D, 19.4% versus LaOT1D, 44.4%; **Supplementary Table 1**). Consistent with prior studies (15, 24, 32), EOT1D patients also displayed heightened humoral reactivity against insulin at diagnosis when compared to LaOT1D subjects (anti-insulin antibodies at diagnosis present in 74.1% versus 36.8%, respectively; **Supplementary Table 1**). Collectively, these data indicate that the EOT1D cohort followed the typical T1D clinical phenotype observed in preschool children, and differed from the LaOT1D cohort.

Conventional analysis of T1D risk conferred by HLA class II in Europeans considers 4 different genotype classes (25, 33). The highest risk is conferred by DR3/DR4 genotypes, followed by DR3/3 and DR4/4 (high risk), and by DR3 or DR4 haplotypes in combination with any other haplotype (DR3/X, DR4/X; intermediate risk), while X/X genotypes are considered low risk (25, 33).

In our cohorts, we observed that 91/97 EOT1D (93.8%) and 85/96 LaOT1D (88.5%) subjects, carried at least one of the two main high-risk haplotypes DR3-DQ2 (DRB1*03:01-DQA1*05:01-DQB1*02:01) or DR4-DQ8 (DRB1*04:01/04:02/04:04/04:05/04:08-DQA1*03:01/03:02-DQB1*03:02) in Caucasians. Of these, 46 EOT1D and 28 LaOT1D patients were homozygous or heterozygous DR3-DQ2/DR4-DQ8, whereas 45 EOT1D and 57 LaOT1D patients harbored only one of these haplotypes (DR3/X or DR4/X).

**Figure 1:**
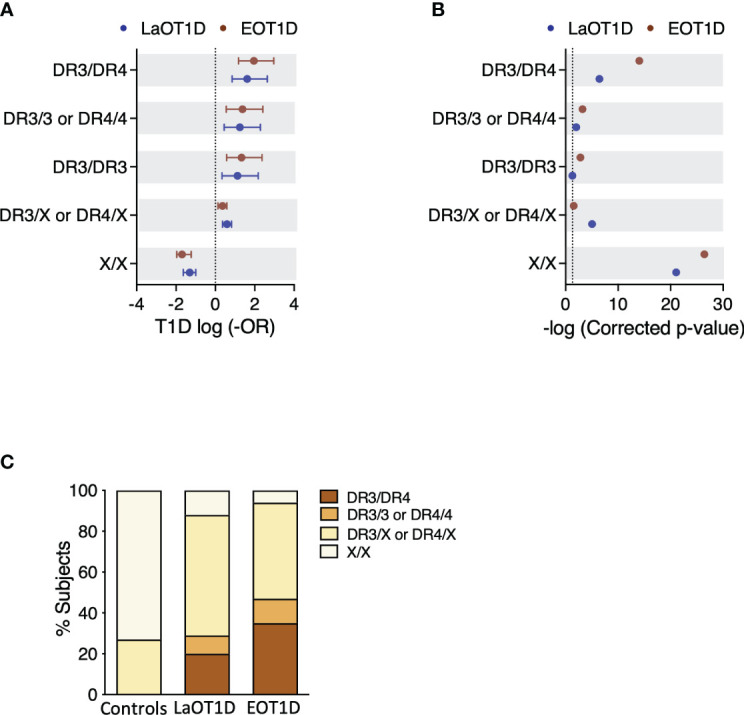
Genetic risk conferred by classical T1D-associated HLA class II genotypes in Early-onset T1D (EOT1D; n=97) and Later-onset T1D patients (LaOT1D; n=96). In **(A)**, the mean log -odds ratio (OR) ± 95% CI of HLA class II individual genotypes in case versus controls (n=169) is shown. EOT1D versus control comparisons are represented in red and LaOT1D versus controls in blue. The dashed line represents log -OR=0. In **(B)**, allelic association tests in case versus controls, represented as -log p-value, after Holm-Bonferroni correction are depicted. The dashed line represents -log p-value=0.05. In **(C)**, the proportion of subjects with the indicated DR3 and DR4 genotypic classes in EOT1D and LaOT1D patients as well as controls are shown (C versus LaOT1D versus EOT1D, p-value<1x10^-3^; LaOT1D versus EOT1D, p-value=5.63x10^-2^; by chi-square test).

We found that disease risk conferred by DR3 and DR4 susceptibility genotypes was not significantly different in EOT1D and LaOT1D cohorts (**Figure 1A** and **Supplementary Table 2**). Likewise, HLA class II genotypes not containing DR3 or DR4 (X/X) had comparable protective effects in the two disease groups (**Figure 1A** and **Supplementary Table 2**). As expected, association analysis detected a significantly increased frequency of DR3 and DR4 genotypes in EOT1D and LaOT1D patients, in comparison with non-diabetic controls (**Figure 1B** and **Supplementary Table 2**) and confirmed that the frequency of risk and protective genotype classes was not significantly different in EOT1D in comparison with LaOT1D subjects (**Supplementary Figure 1**). Moreover, the distribution of the different genotype classes was not significantly different in EOT1D when compared with LaOT1D, while clearly being so when the 3 groups were compared together (**Figure 1C**). Nonetheless, in line with previous studies (15, 23–26, 34) we found a trend for higher frequency of the highest risk genotype (DR3/DR4) in EOT1D patients when compared to LaOT1D subjects (**Supplementary Table 2**, 35.1% versus 19.8%, p-value=2.36x10^-2^, by Fisher´s exact test, prior multi-comparison correction). These results prompted us to dissect the HLA class II genetic configurations in these patient groups to discern whether different alleles in DR3 and DR4 genotype classes show differential association with EOT1D when compared to LaOT1D.

### 3.2 Private and shared HLA class II alleles associated with EOT1D and LaOT1D

**Figure 2:**
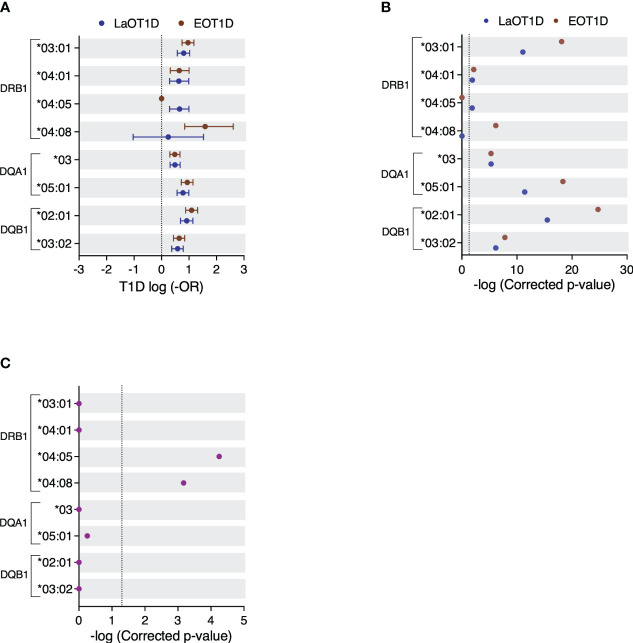
Genetic risk conferred by HLA class II alleles in EOT1D and LaOT1D patients. In **(A)**, the mean log-odds ratio (OR) ± 95% CI of individual HLA class II genotypes in cases versus controls is displayed. Comparisons of EOT1D versus controls are highlighted in red, and LaOT1D versus controls are shown in blue. The dashed line represents log-OR=0. In **(B)**, allelic association tests in cases versus controls, presented as -log p-values after Holm-Bonferroni correction, are depicted. The dashed line represents -log p-value=0.05. In **(C)**, allelic association analysis in EOT1D versus LaOT1D patients (purple). The dashed line represents -log p-value=0.05.

To scrutinize the contributions of different HLA class II genes in disease susceptibility, we analyzed the genetic risk conferred by DRB1, DQA1 and DQB1 alleles with a frequency ≥2.5% in the EOT1D and LaOT1D cohorts or in control subjects. Most risk alleles had comparable genetic effects in EOT1D and LaOT1D cohorts (**Figure 2A** and **Supplementary Table 3**). Interestingly, the T1D risk allele DRB1*04:05 was absent in EOT1D subjects, suggesting it may not significantly contribute to the development of EOT1D (**Figure 2A** and **Supplementary Table 3**). We also noted that the DQA1*05:05 and DQB1*02:02 protective alleles were under-represented in EOT1D when compared to LaOT1D subjects, suggesting that disease protection conferred by these alleles is particularly relevant in EOT1D (**Supplementary Figure 2** and **Supplementary Table 4**).

Strikingly, the DRB1*04:08 allele was significantly associated with EOT1D but not with LaOT1D (**Figure 2B** and **Supplementary Table 3**). Consistent with earlier findings in Portuguese (35, 36) and Spanish cohorts (37), the DRB1*04:08 allele was found at a remarkably low frequency in nondiabetic controls (0.3%, **Supplementary Table 3**). Conversely, we found that the classical T1D risk allele DRB1*04:05 was associated with LaOT1D while it was absent in the EOT1D cohort. Although the absence of the DRB1*04:05 allele in EOT1D patients might appear surprising, it aligns with findings from a previous study involving Caucasian children diagnosed with T1D before the age of five (13). Therefore, we next directly compared allelic frequencies in EOT1D and LaOT1D subjects. This analysis corroborated that the DRB1*04:05 risk allele as well as the DQA1*05:05 and DQB1*02:02 protective alleles have a significantly higher frequency in LaOT1D when compared to EOT1D (**Figure 2C** and **Supplementary Figure 2C**, **Supplementary Tables 3, 4**). However, no difference in the frequency of other risk and protective alleles was observed in LaOT1D when compared to EOT1D (**Figure 2C** and **Supplementary Figure 2C**). Notably, we found that the rare allele DRB1*04:08, while not associated with LaOT1D, was present in 20 of the 97 EOT1D subjects and conferred the highest HLA class II allelic risk to EOT1D (**Figure 2** and **Supplementary Table 3**).

T1D susceptibility conferred by distinct HLA alleles may arise from variations in amino acid residues at specific positions within the peptide-binding groove. We thus performed amino acid sequence alignment of the DRB1*04 alleles analyzed in this study (**Supplementary Figure 3**) and found that the presence of serine (S) instead of aspartic acid (D) at amino acid position 57 was the only difference between the LaOT1D-specific DRB1*04:05 and the EOT1D-associated DRB1*04:08 risk alleles. While D and S are both polar amino acids, D is negatively charged whereas S is uncharged. This distinction in charge within pocket 9 may influence both the nature and the affinity of diabetogenic peptides binding to the MHC molecules encoded by these two alleles.

Previous studies have also established that DQ-susceptible alleles code for arginine (R) residue at position 52 of the DQA1 molecule and are negative for D at the DQB1 position 57 (7, 8). Consistent with these findings, we found that the presence of R in DQA1 position 52 conferred significant risk, whereas its substitution by histidine or serine was protective in EOT1D or in both EOT1D and LaOT1D, respectively (**Supplementary Table 5**). Moreover, while D at position 57 of DQB1 was highly protective, the presence of alanine (A) was associated with significant risk in both cohorts (**Supplementary Table 6**). The representation of susceptible and protective alleles defined by these amino acid residues was not different in EOT1D when compared to LaOT1D (**Supplementary Figure 4**). Together, these findings uncovered that in addition to shared HLA class II allelic variants (DRB1*03:01, DRB1*04:01, DQA1*03, DQA1*05:01, DQB1*02:01 and DQB1*03:02), distinct alleles (DRB1*04:08 and DRB1*04:05) are contributing to the risk of EOT1D or LaOT1D. This led us to compare the HLA class II haplotypic structure in these cohorts, with a focus on DR3 and DR4 risk haplotypes.

### 3.3 EOT1D distinctive HLA class II haplotypes

**Figure 3:**
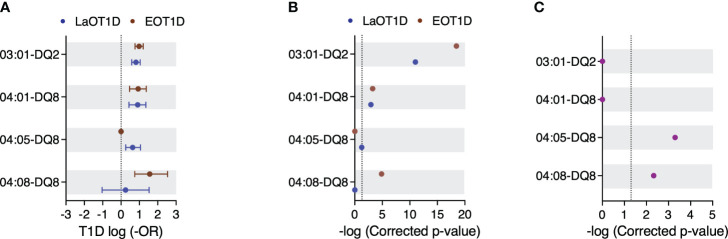
Genetic risk conferred by HLA class II susceptibility haplotypes in EOT1D and LaOT1D patients. In **(A)**, the mean log-odds ratio (OR) ± 95% CI of individual HLA class II genotypes in cases versus controls is illustrated. Comparisons of EOT1D versus controls are highlighted in red, and LaOT1D versus controls are represented in blue. The dashed line represents log -OR=0. In **(B)**, allelic association tests in cases versus controls are presented, depicting -log p-values after Holm-Bonferroni correction. The dashed line represents -log p-value=0.05. In **(C)**, allelic association analysis in EOT1D versus LaOT1D patients (purple). The dashed line represents -log p-value=0.05.

Haplotype reconstruction identified 29, 50 and 55 distinct class II haplotypes in EOT1D, LaOT1D and control subjects, respectively. This analysis revealed 3 protective and 3 risk haplotypes significantly associated with EOT1D and LaOT1D (**Figures 3A, B** and **Supplementary Figure 5** and **Supplementary Tables 7, 8**). The DRB1*15:01-DQA1*01:02-DQB1*06:02 haplotype was under-represented in both patient groups in comparison to control subjects, whereas the DRB1*07:01-DQA1*02:01-DQB1*02:01/02:02 and DRB1*11:01-DQA1*05:05-DQB1*03:01 haplotypes showed a protective effect only in the EOT1D cohort (**Supplementary Figure 5** and **Supplementary Table 8**).

We also observed that the high-risk DRB1*03:01-DQ2 and DRB1*04:01-DQ8 haplotypes were not differently represented in EOT1D and LaOT1D cohorts (**Figure 3C** and **Supplementary Table 7**), suggesting they are shared susceptibility factors in both disease subtypes. Notably, we found that the risk DRB1*04:08 allele exceedingly occurred in the context of the DQ8 haplotype in EOT1D (17/20 subjects), indicating that the DRB1*04:08-DQ8 haplotype is distinctively associated with this disease subtype (**Figure 3** and **Supplementary Table 7**). Of note, of the three additional patients harboring the DRB1*04:08 allele, two carried DQA1*03:01-DQB1*02:01 and one DQA1*03:01-DQB1*02:51; both are likely risk configurations, given the presence of R and a non-D at positions 52 and 57 of DQA1 and DQB1, respectively (7, 8). Moreover, within the 17 EOT1D children carrying the DRB1*04:08-DQ8 haplotype, 10 were DR3/DR4 heterozygous and 7 harbored it together with neutral haplotypes. Worth noting, the DRB1*04:08-DQ8 haplotype was identified in a single LaOT1D patient who had a diagnosis of Juvenile arthritis at 7 years of age and subsequently developed T1D at the age of 14, thus presenting an unusual combination of autoimmune diseases.

The DRB1*04:05 allele also occurred at high frequency in the context of DQ8 in LaOT1D (15/18 subjects; one of these homozygous) but failed to attain statistical significance after multiple testing correction (corrected p-value=5.46x10^-2^; **Figure 3** and **Supplementary Table 7**). Of the three additional carriers of the DRB1*04:05 allele, two presented it associated with DQA1*03:02-DQB1*02:01 and the third with the DQA1*05:01-DQB1*02:01 haplotype. Within the 15 LaOT1D carriers of the DRB1*04:05-DQ8 haplotype, 3 were DR3/DR4 heterozygous, one was homozygous for the haplotype and the others presented it in combination with neutral haplotypes.

It has been reported that the amino acid residues in positions 13 and 71 in the DRB1 molecule and position 57 in DQB1 together capture more than 90% of the phenotypic variance controlled by the HLA locus (9). We found that the A-H-K and A-S-K haplotypes defined by these positions conferred risk in both cohorts (**Supplementary Table 9**). Additionally, while the D-S-R haplotype was protective in both cohorts, D-S-E was protective in EOT1D whereas D-G-R and D-R-A conferred protection in LaOT1D only (**Supplementary Table 9**). Moreover, no differential representation was observed in risk and protective haplotypes defined by these amino acid residues in EOT1D when compared to LaOT1D (**Supplementary Figure 6**).

In summary, these data revealed that in addition to the classical HLA class II susceptibility haplotypes shared between EOT1D and LaOT1D (DRB1*03:01-DQ2 and DRB1*04:01-DQ8), a specific DR4 allelic variant, DRB1*04:08, mostly occurring in the context of the DQ8 haplotype, is distinctively associated with EOT1D and confers the highest risk among the HLA class II haplotypes represented in this cohort.

### 3.4 The DRB1*04:08-DQ8 is the main HLA class II haplotype discriminating EOT1D

**Figure 4:**
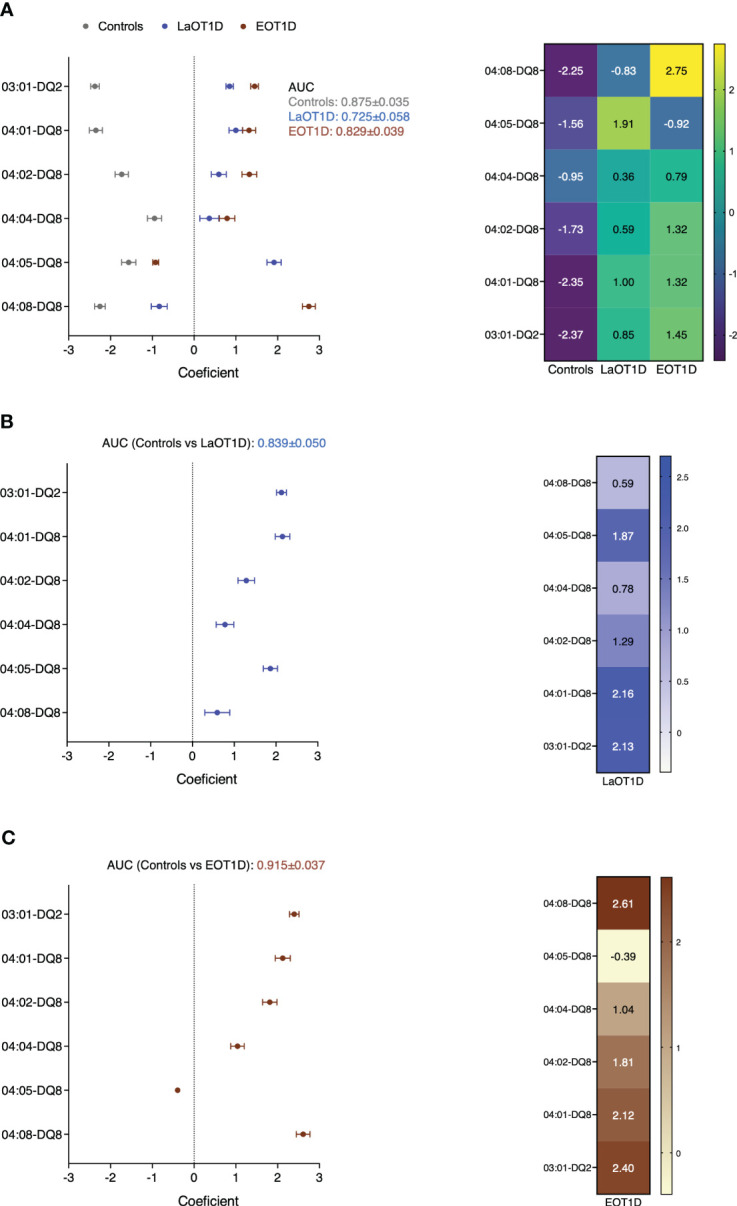
Phenotype predictive power of HLA class II DR3 and DR4 haplotypes in EOT1D and LaOT1D. Coefficients of regularized logistic regression are represented with error bars for each phenotype class. AUC was derived from ROC analysis of one versus the rest in **(A)**, or case group vs controls in **(B, C)** after fitting a binary logistic regression model.

To estimate the predictive power of the DR3 and DR4 risk haplotypes in EOT1D and LaOT1D classification, we performed regularized logistic regression with cross-validation, using all three groups simultaneously (each one versus the other two). This analysis revealed that the combined effect of DR3-DQ2 and DR4-DQ8 haplotypes provided solid discrimination between the 3 groups of subjects (Area Under the Curve, AUC, HC: 0.875 ± 0.035; LaOT1D: 0.725 ± 0.058; EOT1D: 0.829 ± 0.039), with the DRB1*04:08-DQ8 haplotype accounting for the highest genetic difference between EOT1D and the other two groups (LaOT1D and controls). Similarly, the DRB1*04:05-DQ8 haplotype was the main contributor distinguishing LaOT1D from the other two cohorts (**Figure 4A**). Furthermore, binary logistic regression results (case group versus controls) aligned with the case-control findings above-mentioned. Accordingly, we found that the DRB1*03:01-DQ2 and DRB1*04:01-DQ8 haplotypes are the main risk factors in LaOT1D (**Figure 4B**), with an associated OR of 8.43 and 8.63, respectively (**Supplementary Table 10**), whereas the DRB1*04:08-DQ8 haplotype is the predictor variable with the highest impact in EOT1D development (OR 13.62; **Figure 4C** and **Supplementary Table 10**).

**Figure 5:**
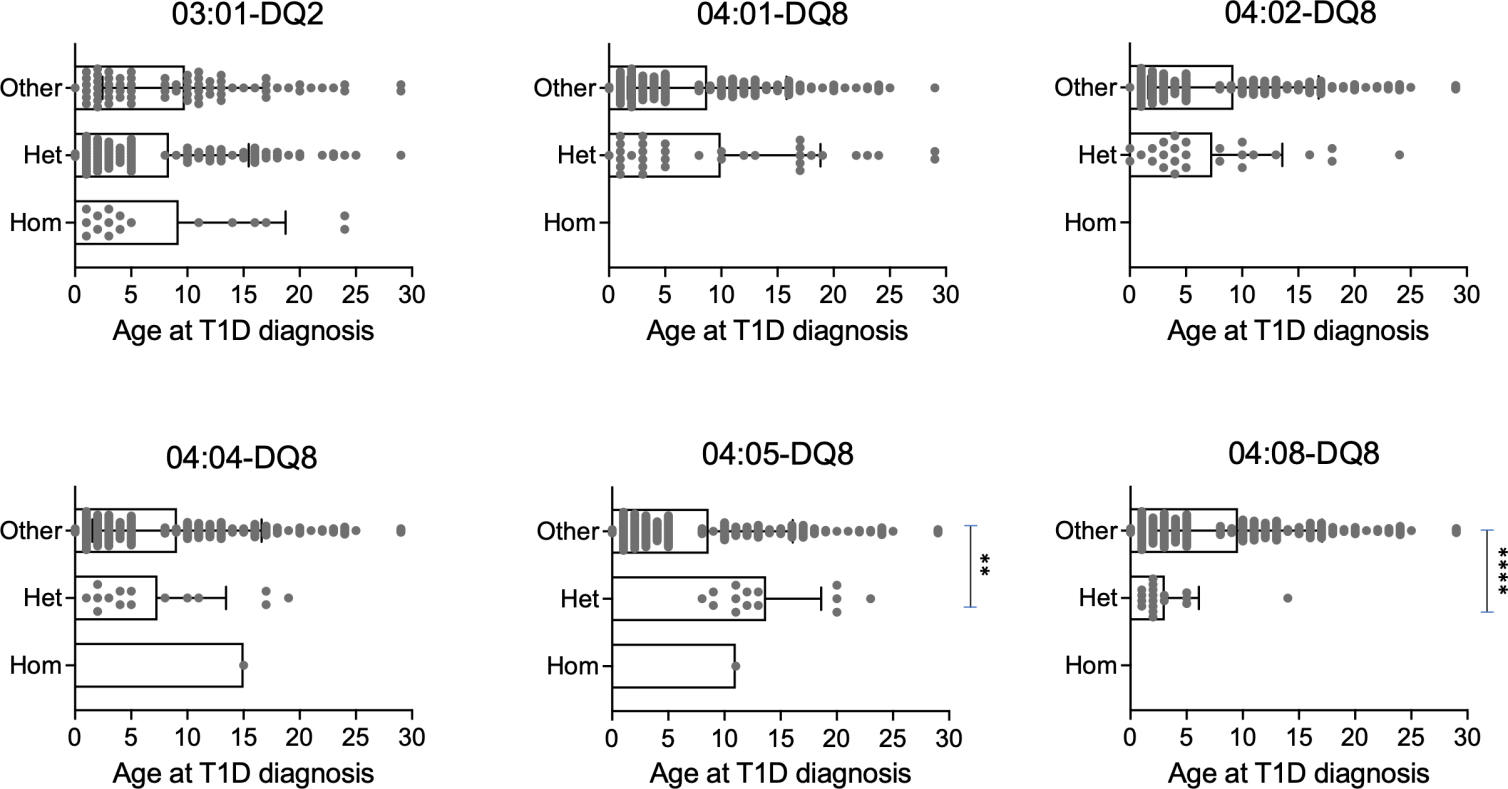
Genotypic stratification of age of T1D onset in patients (n=193) stratified by HLA class II haplotypes. Age distribution of homozygous and heterozygous subjects for DR3 and the indicated DR4 haplotypes are plotted against all other haplotypes (****p=6.29x10^-5^, **p=4.20x10^-3^, by Mann-Whitney test).

We next evaluated whether these haplotypes influenced age at disease onset in all T1D subjects enrolled in this study (n=193). We found no significant impact of protective haplotypes as well as of the DR3/DR4 genotype on age at disease presentation in T1D subjects (**Supplementary Figures 7**, **8**). Importantly, we observed that T1D patients harboring one copy of the DRB1*04:05-DQ8 risk haplotype were on average 13.7 years of age at T1D onset whereas the age at diagnosis in individuals with other haplotypes decreased to 8.6 years. Conversely, subjects with one copy of the DRB1*04:08-DQ8 haplotype were on average 3.1 years of age at disease onset while individuals carrying other haplotypes were 9.6 years old (**Figure 5**). This analysis demonstrated that the discriminative EOT1D and LaOT1D haplotypes significantly influenced the age at T1D presentation, with the DRB1*04:08-DQ8 haplotype decreasing age at onset on average by 6.5 years and the DRB1*04:05-DQ8 haplotype increasing it by 5.1 years.

In sum, while our cohorts of EOT1D and LaOT1D share the expected DQ risk alleles and haplotypes, including DR3-DQ2 and DR4:01-DQ8 (4–6, 35, 37), we revealed the differential impact of two risk DR4 haplotypes, DRB1*04:05-DQ8 and DRB1*04:08-DQ8, and identify the latter as the main single HLA class II discriminative factor and genetic driver in the development of EOT1D, in Portuguese subjects.

## 4 Discussion

In this study, we addressed the question of whether patients with EOT1D harbor a distinctive HLA class II genetic spectrum. Compared with patients with later-onset disease and non-diabetic controls, we found that a rare HLA class II haplotype (DRB1*04:08-DQ8) was primarily present in EOT1D and accounted for the main HLA class II discriminative factor and genetic driver of this T1D subtype. Analysis of age at diagnosis in all T1D subjects confirmed that the DRB1*04:08-DQ8 haplotype was significantly associated with disease onset at younger ages, while the DRB1*04:05-DQ8 haplotype, a main risk DR4 haplotype in Caucasians (6), was absent in EOT1D and associated with diagnosis at older ages. These findings corroborate the notion that specific HLA class II configurations are relevant drivers in determining T1D disease subtypes.

Our study design used an enrollment strategy that targeted subjects who developed T1D at an early age (under 6 years) thereby enriching for genetic susceptibility factors underlying this condition. This approach is in contrast with most studies that evaluated the genetic susceptibility conferred by HLA class II alleles, haplotypes and genotypes within T1D cohorts and families (4–6, 35, 37), where EOT1D subjects are often a minority group. It is thus not surprising that rare alleles and haplotypes, such as DRB1*04:08 and its DRB1*04:08-DQ8 haplotype, may have passed unnoticed in these studies.

In this study, our choice of a healthy control cohort deviates from the gold standard case-control analysis as these individuals are not age-matched with the patient populations (EOT1D and LaOT1D). However, this decision aligns with the study’s specific focus on uncovering specific HLA II genetic determinants of EOT1D. By opting for healthy older controls (IQR [35-51] years), representative of the Portuguese population, we aimed to ensure their unlikely development of T1D during the study, making them an ideal group for identifying HLA II alleles and haplotypes associated with T1D susceptibility. One potential limitation of our control selection strategy arises from the known accumulation of somatic mutations with age. These mutations can act as confounding factors in genetic association studies, particularly when *de novo* variants confer a significant clonal advantage. Somatic mutations in HLA genes have been identified in patients with solid and hematological cancers (38, 39); in hematological cancer recipients of allogeneic and haploidentical stem cell transplantation (39, 40); and in patients with aplastic anemia (41). The significance of these mutations lies in their provision of a selective advantage to the mutated clones, enabling them to evade immune surveillance. An increased accumulation of somatic mutations with age can also be found in healthy individuals (a phenomenon designated by age-related clonal hematopoiesis), but these primarily target genes with malignancy implications, such as *DNMT3A*, *TET2*, and *ASXL1*, and increase their carriers´ predisposition to hematological malignancies and cardiovascular disease (42–45). These mutations are, in addition, individually very rare. Consequently, while we do not rule out the possibility of somatic mutations being present, at least in some of our healthy controls, we consider it improbable that these mutations would specifically target HLA class II genes at a frequency substantial enough to significantly impact our association analysis and the conclusions derived from it.

Our findings bear consequences on age at disease onset. Considering the increasing incidence of EOT1D in the last decades and a prevalence of T1D in the general population of 0.4% (46), along with the assumption that EOT1D presently accounts for 25% of all T1D cases, the application of Bayes theorem predicts that one individual with the DRB1*04:08-DQ8 haplotype has a 3.15% probability of developing EOT1D. On the other hand, the chance of developing T1D at an older age (LaOT1D) is roughly 6 times lower (0.54%). Regarding the DRB1*04:05-DQ8 haplotype, a carrier has 1.18% probability of developing LaOT1D and is unlikely to develop EOT1D.

The DRB1*04:08 allele and the DRB1*04:08-DQ8 haplotype were not found associated with T1D in prior cohort analyses of Portuguese and Spanish T1D patients nor in large Caucasian cohorts (4–6, 35, 37). To the best of our knowledge, this allele was only found significantly overrepresented in T1D patients in comparison with controls in eastern Baltic individuals, when in combination with DQB1*03:04 (47). However, the DRB1*04:08 allele has been previously associated with other autoimmune conditions and associated clinical phenotypes, namely anti-citrullinated protein antibody-positive and childhood Rheumatoid Arthritis (RA), clinical severity of RA and anti-drug antibody development in Multiple Sclerosis patients under Interferon beta treatment (48–50). These observations raise the possibility the DRB1*04:08 allele impacts the development, age at onset and clinical severity of autoimmunity phenotypes other than T1D.

Other studies reported lower frequency of HLA class II protective alleles and haplotypes in patients with T1D onset at a younger age (13, 22, 23, 34). In agreement with these reports, our EOT1D cohort lacked the DRB1*11:01-DQA1*05:05-DQB1*03:01 haplotype and had a significantly lower frequency of the DRB1*07:01-DQA1*02:01-DQB1*02:01/02:01 in comparison with controls, suggesting these may confer protection from early-onset disease, while not impacting significantly on LaOT1D. On the other hand, the classical DRB1*15:01-DQA1*01:02-DQB1*06:02 protective haplotype showed strong effects in both cohorts. These data also raise the possibility that the protective effects of HLA class II haplotypes may differ in these disease subtypes.

Previous studies demonstrated T1D patients diagnosed at a young age present with a more restricted range of DR and DQ haplotypes (24, 34). Accordingly, we found that the HLA class II haplotypic diversity was significantly more homogeneous in EOT1D (29 haplotypes identified) than LaOT1D (50 haplotypes identified; p-value=8.0x10^-3^, Fisher´s exact test). This restriction of haplotypic heterogeneity is likely influenced by the decreased number of DRB1 alleles in EOT1D (19 versus 27 alleles in EOT1D and LaOT1D, respectively), as the allelic heterogeneity in DQA1 (7 versus 9 alleles) and DQB1 (13 versus 12 alleles) is rather similar in the two cohorts. It is conceivable that the limited spectrum of DRB1 susceptibility alleles represents a distinctive feature of EOT1D, with probable impact on CD4 T cell repertoire selection in the thymus, including regulatory T cells, as well as in the activation of these cells in the periphery. Particularly relevant in this context will be to evaluate the binding affinity of distinct diabetogenic peptides to DRB1*04:05-DQ8 compared to DRB1*04:08-DQ8, as it is known that the presence of a non-D (S)/D residue at position 57 within pocket 9 determines the peptide anchor residue accommodated in this pocket (acidic versus small aliphatic) (51, 52).

## 5 Conclusion

Despite the limited size of the cohorts analyzed here, our data suggest EOT1D is a clinical entity bearing non-overlapping genetic determinants when compared to LaOT1D. The distinctive HLA class II differences we revealed may be relevant in the implementation of EOT1D screening strategies and personalized therapeutic approaches, such as peptide-based immunotherapy. Multicentric studies with broader ethnic coverage would be helpful in replicating these findings and in identifying additional private EOT1D genetic factors, that may be of use as predictors of age at T1D onset and disease severity.

## Data availability statement

The datasets presented in this article are not readily available because we do not have ethical approval for depositing the raw genotype information related to these children in online public repositories. Requests to access the datasets should be directed to the corresponding author.

## Ethics statement

The studies involving humans were approved by Ethics Committee from Hospital de Dona Estefânia (HDE; Comissão de Ética para a Saúde, Centro Hospital de Lisboa Central, #318/2016), and the Ethics Committee from Associação Protetora dos Diabéticos de Portugal (APDP). The studies were conducted in accordance with the local legislation and institutional requirements. Written informed consent for participation in this study was provided by the participants or participants’ legal guardians/next of kin.

## Author contributions

IC: Conceptualization, Formal analysis, Funding acquisition, Investigation, Project administration, Supervision, Validation, Visualization, Writing – original draft. PM: Data curation, Investigation, Writing – review & editing. DL: Investigation, Writing – review & editing. TP: Formal analysis, Methodology, Software, Writing – review & editing. DS: Formal analysis, Software, Writing – review & editing. AF: Data curation, Resources, Writing – review & editing. CL: Conceptualization, Data curation, Resources, Writing – review & editing. JD: Conceptualization, Funding acquisition, Validation, Writing – original draft. CP-G: Conceptualization, Funding acquisition, Validation, Writing – original draft.

## References

[1] Patterson CC Dahlquist G Soltész G Green A. Variation and trends in incidence of childhood diabetes in Europe. Lancet (2000) 355:873–6. doi: 10.1016/S0140-6736(99)07125-1 10752702

[2] Karvonen M. Incidence and trends of childhood Type 1 diabetes worldwide 1990–1999. Diabetic Med (2006) 23:857–66. doi: 10.1111/J.1464-5491.2006.01925.X 16911623

[3] Noble JA Valdes AM Cook M Klitz W Thomson G Erlich HA. The role of HLA class II genes in insulin-dependent diabetes mellitus: molecular analysis of 180 Caucasian, multiplex families. Am J Hum Genet (1996) 59:1134–48. PMC1914851 8900244

[4] Lambert AP Gillespie KM Thomson G Cordell HJ Todd JA Gale EAM. Absolute risk of childhood-onset type 1 diabetes defined by human leukocyte antigen class II genotype: A population-based study in the United Kingdom. J Clin Endocrinol Metab (2004) 89:4037–43. doi: 10.1210/jc.2003-032084 15292346

[5] Hermann R Turpeinen H Laine AP Veijola R Knip M Simell O. HLA DR-DQ-encoded genetic determinants of childhood-onset type 1 diabetes in Finland: An analysis of 622 nuclear families. Tissue Antigens (2003) 62:162–9. doi: 10.1034/J.1399-0039.2003.00071.X 12889996

[6] Erlich H Valdes AM Noble J Carlson JA Varney M Concannon P. HLA DR-DQ haplotypes and genotypes and type 1 diabetes risk analysis of the type 1 diabetes genetics consortium families. Diabetes (2008) 57:1084–92. doi: 10.2337/db07-1331 PMC4103420 18252895

[7] Todd JA Bell JI McDevitt HO. HLA-DQβ gene contributes to susceptibility and resistance to insulin-dependent diabetes mellitus. Nature (1987) 329:599–604. doi: 10.1038/329599a0 3309680

[8] Khalil I d’Auriol L Gobet M Morin L Lepage V Deschamps I. A combination of HLA-DQβ Asp57-negative and HLA DQα Arg52 confers susceptibility to insulin-dependent diabetes mellitus. J Clin Invest (1990) 85:1315–9. doi: 10.1172/JCI114569 PMC296568 2318983

[9] Hu X Deutsch AJ Lenz TL Onengut-Gumuscu S Han B Chen WM. Additive and interaction effects at three amino acid positions in HLA-DQ and HLA-DR molecules drive type 1 diabetes risk. Nat Genet (2015) 47:898–905. doi: 10.1038/ng.3353 26168013 PMC4930791

[10] Onengut-Gumuscu S Chen WM Burren O Cooper NJ Quinlan AR Mychaleckyj JC. Fine mapping of type 1 diabetes susceptibility loci and evidence for colocalization of causal variants with lymphoid gene enhancers. Nat Genet (2015) 47:381–6. doi: 10.1038/ng.3245 PMC4380767 25751624

[11] Shapiro MR Thirawatananond P Peters L Sharp RC Ogundare S Posgai AL. De-coding genetic risk variants in type 1 diabetes. Immunol Cell Biol (2021) 99:496–508. doi: 10.1111/imcb.12438 33483996 PMC8119379

[12] Leete P Willcox A Krogvold L Dahl-Jørgensen K Foulis AK Richardson SJ. Differential insulitic profiles determine the extent of β-cell destruction and the age at onset of type 1 diabetes. Diabetes (2016) 65:1362–9. doi: 10.2337/db15-1615 26858360

[13] Hathout EH Hartwick N Fagoaga OR Colacino AR Sharkey J Racine M. Clinical, autoimmune, and HLA characteristics of children diagnosed with type 1 diabetes before 5 years of age. Pediatrics (2003) 111:860–3. doi: 10.1542/peds.111.4.860 12671124

[14] Kuhtreiber WM Washer SLL Hsu E Zhao M Reinhold P Burger D. Low levels of C-peptide have clinical significance for established Type 1 diabetes. Diabetes Med (2015) 32:1346–53. doi: 10.1111/DME.12850 PMC4578991 26172028

[15] Komulainen J Kulmala P Savola K Lounamaa R Ilonen J Reijonen H. Clinical, autoimmune, and genetic characteristics of very young children with type 1 diabetes. Childhood Diabetes in Finland (DiMe) Study Group. Diabetes Care (1999) 22:1950–5. doi: 10.2337/DIACARE.22.12.1950 10587824

[16] Rawshani A Sattar N Franzén S Rawshani A Hattersley AT Svensson AM. Excess mortality and cardiovascular disease in young adults with type 1 diabetes in relation to age at onset: a nationwide, register-based cohort study. Lancet (2018) 392:477–86. doi: 10.1016/S0140-6736(18)31506-X PMC6828554 30129464

[17] Fava D Gardner S Pyke D David R Leslie G. Evidence that the age at diagnosis of IDDM is genetically determined. Diabetes Care (1998) 21:925–9. doi: 10.2337/diacare.21.6.925 9614609

[18] Valdes AM Thomson G Erlich HA Noble JA. Association between type 1 diabetes age of onset and HLA among sibling pairs. Diabetes (1999) 48:1658–61. doi: 10.2337/diabetes.48.8.1658 10426387

[19] Howson JMM Rosinger S Smyth DJ Boehm BO Aldinger G Aufschild J. Genetic analysis of adult-onset autoimmune diabetes. Diabetes (2011) 60:2645–53. doi: 10.2337/db11-0364 PMC3178303 21873553

[20] Howson JMM Cooper JD Smyth DJ Walker NM Stevens H She JX. Evidence of gene-gene interaction and age-at-diagnosis effects in type 1 diabetes. Diabetes (2012) 61:3012–7. doi: 10.2337/db11-1694 PMC3478521 22891215

[21] Inshaw JRJ Walker NM Wallace C Bottolo L Todd JA. The chromosome 6q22.33 region is associated with age at diagnosis of type 1 diabetes and disease risk in those diagnosed under 5 years of age. Diabetologia (2018) 61:147–57. doi: 10.1007/s00125-017-4440-y PMC5719131 28983737

[22] Inshaw JRJ Cutler AJ Crouch DJM Wicker LS Todd JA. Genetic variants predisposing most strongly to type 1 diabetes diagnosed under age 7 years lie near candidate genes that function in the immune system and in pancreatic B-cells. Diabetes Care (2020) 43:169–77. doi: 10.2337/dc19-0803 PMC6925581 31558544

[23] Caillat-Zucman S Garchon HJ Timsit J Assan R Boitard C Djilali- Saiah I. Age-dependent HLA genetic heterogeneity of type 1 insulin-dependent diabetes mellitus. J Clin Invest (1992) 90:2242–50. doi: 10.1172/JCI116110 PMC443375 1469084

[24] Tait BD Harrison LC Drummond BP Stewart V Varney MD Honeyman MC. HLA antigens and age at diagnosis of insulin-dependent diabetes mellitus. Hum Immunol (1995) 42:116–22. doi: 10.1016/0198-8859(94)00075-2 7744614

[25] Gillespie KM Gale EAM Bingley PJ. High familial risk and genetic susceptibility in early onset childhood diabetes. Diabetes (2002) 51:210–4. doi: 10.2337/diabetes.51.1.210 11756343

[26] Emery L Babu S Bugawan T Norris J Erlich H Eisenbarth G. Newborn HLA-DR, DQ genotype screening: age-and ethnicity-specific type 1 diabetes risk estimates. Pediatr Diabetes (2005) 6:136–44. doi: 10.1111/j.1399-543X.2005.00117.x PMC1351310 16109069

[27] American Diabetes Association. Diagnosis and classification of diabetes mellitus. Diabetes Care (2014) 37:S81–S90. doi: 10.2337/dc14-S081 24357215

[28] Patarrão RS Duarte N Coelho I Ward J Ribeiro RT Meneses MJ. Prediabetes blunts DPP4 genetic control of postprandial glycaemia and insulin secretion. Diabetologia (2022) 65:861–71. doi: 10.1007/s00125-021-05638-6 PMC8960640 35190847

[29] Excoffier L Lischer HEL. Arlequin suite ver 3.5: A new series of programs to perform population genetics analyses under Linux and Windows. Mol Ecol Resour (2010) 10:564–7. doi: 10.1111/j.1755-0998.2010.02847.x 21565059

[30] Pedregosa F Varoquaux G Gramfort A Michel V Thirion B Grisel O. Scikit-learn: machine learning in python. J Mach Learn Res (2011) 12:2825–30.

[31] Couper JJ Hudson I Werther GA Warne GL Court JM Harrison LC. Factors predicting residual β-cell function in the first year after diagnosis of childhood type 1 diabetes. Diabetes Res Clin Pract (1991) 11:9–16. doi: 10.1016/0168-8227(91)90135-Z 2019237

[32] Sales Luis M Alcafache M Ferreira S Fitas AL Simões Pereira J Caramalho Í. Children with type 1 diabetes of early age at onset - Immune and metabolic phenotypes. J Pediatr Endocrinol Metab (2019) 32:935–41. doi: 10.1515/jpem-2019-0103 31280235

[33] Gillespie KM Bain SC Barnett PAH Bingley PPJ Christie MR Gill GV. The rising incidence of childhood type 1 diabetes and reduced contribution of high-risk HLA haplotypes. Lancet (2004) 364:1699–700. doi: 10.1016/S0140-6736(04)17357-1 15530631

[34] Reinauer C Rosenbauer J Bächle C Herder C Roden M Ellard S. The clinical course of patients with preschool manifestation of type 1 diabetes is independent of the HLA DR-DQ genotype. Genes (Basel) (2017) 8:146–6. doi: 10.3390/genes8050146 PMC5448020 28534863

[35] Spínola H Lemos A Couto AR Parreira B Soares M Dutra I. Human leucocyte antigens class II allele and haplotype association with Type 1 Diabetes in Madeira Island (Portugal). Int J Immunogenet (2017) 44:305–13. doi: 10.1111/iji.12335 28834219

[36] Armas J Couto R Santos M Bettencourt B. HLA-A, -B, -Cw, -DQA1, -DQB1 and -DRB1 alleles in a population from the Azores. Hum Immunol (2004) 65:862–4. doi: 10.1016/j.humimm.2004.08.006

[37] Escribano-De-Diego J Sánchez-Velasco P Luzuriaga C Ocejo-Vinyals JG Paz-Miguel JE Leyva-Cobián F. HLA class II immunogenetics and incidence of insulin-dependent diabetes mellitus in the population of Cantabria (Northern Spain). Hum Immunol (1999) 60:990–1000. doi: 10.1016/S0198-8859(99)00077-4 10566601

[38] Shukla SA Rooney MS Rajasagi M Tiao G Dixon PM Lawrence MS. Comprehensive analysis of cancer-associated somatic mutations in class i HLA genes. Nat Biotechnol (2015) 33:1152–8. doi: 10.1038/nbt.3344 PMC4747795 26372948

[39] Smith AG Fan W Regen L Warnock S Sprague M Williams R. Somatic mutations in the HLA genes of patients with hematological Malignancy. Tissue Antigens (2012) 79:359–6. doi: 10.1111/j.1399-0039.2012.01868.x 22489945

[40] Pagliuca S Gurnari C Hercus C Hergalant S Hong S Dhuyser A. Leukemia relapse *via* genetic immune escape after allogeneic hematopoietic cell transplantation. Nat Commun (2023) 14:3153. doi: 10.1038/s41467-023-38113-4 37258544 PMC10232425

[41] Babushok DV Duke JL Xie HM Stanley N Atienza J Perdigones N. Somatic HLA mutations expose the role of class I-mediated autoimmunity in aplastic anemia and its clonal complications. Blood Adv (2017) 1:1900–10. doi: 10.1182/bloodadvances.2017010918 PMC5621748 28971166

[42] Jaiswal S Fontanillas P Flannick J Manning A Grauman PV Mar BG. Age-related clonal hematopoiesis associated with adverse outcomes. New Engl J Med (2014) 371:2488–98. doi: 10.1056/nejmoa1408617 PMC4306669 25426837

[43] Genovese G Kähler AK Handsaker RE Lindberg J Rose SA Bakhoum SF. Clonal hematopoiesis and blood-cancer risk inferred from blood DNA sequence. New Engl J Med (2014) 371:2477–87. doi: 10.1056/nejmoa1409405 PMC4290021 25426838

[44] Bick AG Weinstock JS Nandakumar SK Fulco CP Bao EL Zekavat SM. Inherited causes of clonal haematopoiesis in 97,691 whole genomes. Nature (2020) 586:763–8. doi: 10.1038/s41586-020-2819-2 PMC7944936 33057201

[45] Jaiswal S Natarajan P Silver AJ Gibson CJ Bick AG Shvartz E. Clonal hematopoiesis and risk of atherosclerotic cardiovascular disease. New Engl J Med (2017) 377:111–21. doi: 10.1056/nejmoa1701719 PMC6717509 28636844

[46] Redondo M Steck A Pugliese A. Genetics of type 1 diabetes. Pediatr Diabetes (2018) 19:346–53. doi: 10.1111/pedi.12597 PMC5918237 29094512

[47] Ilonen J Koskinen S Nejentsev S Sjöroos M Knip M Schwartz EI. HLA-DQB1(*)0304-DRB1(*)0408 haplotype associated with insulin-dependent diabetes mellitus in populations in the eastern Baltic region. Tissue Antigens (1997) 49:532–4. doi: 10.1111/j.1399-0039.1997.tb02794.x 9174152

[48] Weyand CM Hicok KC Conn DL Goronzy JJ. The influence of HLA-DRB1 genes on disease severity in rheumatoid arthritis. Ann Intern Med (1992) 117:801–6. doi: 10.7326/0003-4819-117-10-801 1416553

[49] Hoffmann S Cepok S Grummel V Lehmann-Horn K Hackermueller J Stadler PF. HLA-DRB1*0401 and HLA-DRB1*0408 are strongly associated with the development of antibodies against interferon-β Therapy in multiple sclerosis. Am J Hum Genet (2008) 83:219–27. doi: 10.1016/j.ajhg.2008.07.006 PMC2495071 18656179

[50] Prahalad S Thompson SD Conneely KN Jiang Y Leong T Prozonic J. Hierarchy of risk of childhood-onset rheumatoid arthritis conferred by HLA-DRB1 alleles encoding the shared epitope. Arthritis Rheum (2012) 64:925–30. doi: 10.1002/art.33376 PMC3276774 21953520

[51] Stern LJ Brown JH Jardetzky TS Gorga JC Urban RG Strominger JL. Crystal structure of the human class II MHC protein HLA-DR1 complexed with an influenza virus peptide. Nature (1994) 368:215–21. doi: 10.1038/368215a0 8145819

[52] Friede T Gnau V Jung G Keilholz W Stevanović S Rammensee HG. Natural ligand motifs of closely related HLA-DR4 molecules predict features of rheumatoid arthritis associated peptides. Biochim Biophys Acta Mol Basis Dis (1996) 1316:85–101. doi: 10.1016/0925-4439(96)00010-5 8672555

