# Supplementary material for: The rare DRB1*04:08-DQ8 haplotype is the main HLA class II genetic driver and discriminative factor of Early-onset Type 1 diabetes in the Portuguese population
Source: Front Immunol. 2024 Jan 3;14:1299609. doi: 10.3389/fimmu.2023.1299609 (PMC10839680; doi:10.3389/fimmu.2023.1299609)
Supplement: Supplementary file 1 [file Presentation_1.pdf]

Supplementary Data

The rare DRB1*04:08-DQ8 haplotype is the main HLA class II genetic driver and discriminative factor of Early-Onset Type 1 Diabetes in the Portuguese population

Iris Caramalho, Paula Matoso, Dário Ligeiro, Tiago Paixão, Daniel Sobral, Ana Laura Fitas, Catarina Limbert, Jocelyne Demengeot and Carlos Penha-Gonçalves

Contained Files: Tables S1 to S10; Figures S1 to S8

Table S1: Clinical characteristics of the patient groups.

	LaOT1D	EOT1D	p-value
T1D first relatives<br>n; nt	8; 38	13; 97	NS [a]
C-peptide at diagnosis<br>(mean±sem, ng/ml; [IQR]; nt)	0.428±0.05; [0.20-0.60]; 32	0.326±0.06; [0.10-0.30]; 51	5.90×10^{-03} [b]
AIA at diagnosis<br>n; nt	25; 68	60; 81	6.01×10^{-06} [a]
%HbA1c at diagnosis<br>(mean±sem; [IQR]; nt)	11.45±0.45; [9.50-13.60]; 34	10.58±0.20; [9.40-11.60]; 69	4.46×10^{-02} [b]
%HbA1c 1Y<br>(mean±sem; [IQR]; nt)	8.14±0.33; [7.20-8.6]; 31	8.47±0.11; [7.80-9.28]; 80	5.60×10^{-03} [b]
%HbA1c 1Y<7.5<br>n; nt	12; 27	12; 62	1.99×10^{-02} [a]

n: number of individuals with the indicated clinical characteristic; nt: number of individuals included in the analysis; sem: standard error of the mean; AIA: anti-insulin autoantibodies; HbA1C: Glycated haemoglobin; HbA1C 1Y: Glycated haemoglobin 1 year after diagnosis; NS, non significant; p-value calculated using Fisher´s exact test [a] or two-tailed Mann-Whitney test [b].

Table S2: Susceptibility and protective HLA class II genotypes in EOT1D and LaOT1D.

Genotypes	Controls n (%)	LaOT1D n (%)	EOT1D n (%)	Subject group	OR [95% CI]	p-value	p-value (corrected)
DR3/DR4	0 (0)	19 (19.8)	34 (35.1)	LaOT1D	>41.45 [7.11-435.40]	$1.69x10^{-08}$	**$3.55x10^{-07}$**
				EOT1D	>90.67 [14.97-933.0]	$4.06x10^{-16}$	**$8.92x10^{-15}$**
DR3/3 or DR4/4	0 (0)	9 (9.4)	12 (12.4)	LaOT1D	>17.38 [2.77-191.80]	$5.81x10^{-04}$	**$9.88x10^{-03}$**
				EOT1D	>23.72 [3.58-255.80]	$3.14x10^{-05}$	**$5.98x10^{-04}$**
DR3/DR3	0 (0)	7 (7.3)	11 (11.3)	LaOT1D	>13.21 [2.17-149.60]	$3.96x10^{-03}$	NS
				EOT1D	>21.49 [3.72-233.20]	$8.60x10^{-05}$	**$1.55x10^{-03}$**
DR4/DR4	0 (0)	2 (2.1)	1 (1.0)	LaOT1D	>3.57 [0.41-52.06]	NS	NS
				EOT1D	>1.75 [0.09-33.42]	NS	NS
DR3/X or DR4/X	46 (27.2)	57 (59.4)	45 (46.4)	LaOT1D	3.91 [2.29-6.63]	$4.58x10^{-07}$	**$9.16x10^{-06}$**
				EOT1D	2.31 [1.36-3.83]	$1.97x10^{-03}$	**$2.96x10^{-02}$**
DR3/X	22 (13.0)	27 (28.1)	22 (22.7)	LaOT1D	2.61 [1.41-5.0]	$3.02x10^{-03}$	**$4.23x10^{-02}$**
				EOT1D	1.96 [1.03-3.65]	NS	NS
DR4/X	24 (14.2)	30 (31.2)	23 (23.7)	LaOT1D	2.75 [1.47-5.01]	$1.39x10^{-03}$	**$2.22x10^{-02}$**
				EOT1D	1.88 [0.98-3.57]	NS	NS
X/X	123 (72.8)	11 (11.5)	6 (6.2)	LaOT1D	0.05 [0.02-0.10]	$3.83x10^{-23}$	**$8.81x10^{-22}$**
				EOT1D	0.02 [0.01-0.06]	$1.56x10^{-28}$	**$3.75x10^{-27}$**

n (%): number and frequency of genotypes in 169 control subjects, 96 LaOT1D subjects and 97 EOT1D subjects. OR: Odds ratio; CI: Confidence interval; p-value, uncorrected; p-value (corrected), Holm-Bonferroni multi-comparison correction. Significant results are depicted in bold. NS, non significant. For genotypes absent in HC, the value of n=1 was considered to calculate the OR.
DR3: DRB1*03:01-DQA1*05:01-DQB1*02:01 haplotype.
DR4: DRB1*04:01/04:02/04:04/04:05/04:08-DQA1*03:01/03:02-DQB1*03:02 haplotypes.
X: other haplotypes than DR3 or DR4.

Table S3: HLA-DRB1, HLA-DQA1 and HLA-DQB1 susceptibility alleles in EOT1D and LaOT1D.

Alleles	Controls n (%)	LaOT1D n (%)	EOT1D n (%)	Subject group	OR [95% CI]	p-value	p-value (corrected)
DRB1*03:01	23 (6.8)	61 (31.8)	78 (40.2)	LaOT1D	6.38 [3.77-10.56]	$1.50x10^{-13}$	**$8.39x10^{-12}$**
				EOT1D	9.21 [5.58-15.01]	$1.34x10^{-20}$	**$7.64x10^{-19}$**
DRB1*04:01	9 (2.7)	20 (10.4)	21 (10.8)	LaOT1D	4.25 [1.96-9.75]	$2.63x10^{-04}$	**$1.26x10^{-02}$**
				EOT1D	4.44 [2.08-10.09]	$1.44x10^{-04}$	**$7.05x10^{-03}$**
DRB1*04:02	8 (2.4)	12 (6.2)	15 (7.7)	LaOT1D	2.75 [1.18-6.58]	$3.19x10^{-02}$	NS
				EOT1D	3.46 [1.49-7.88]	$6.44x10^{-03}$	NS
DRB1*04:04	6 (1.8)	10 (5.2)	9 (4.6)	LaOT1D	3.04 [1.04-8.36]	$3.43x10^{-02}$	NS
				EOT1D	2.69 [1.00-7.60]	NS	NS
DRB1*04:05	8 (2.4)	19 (9.9)	0 (0)	LaOT1D	4.53 [1.96-9.90]	$2.92x10^{-04}$	**$1.37x10^{-02}$**
				EOT1D	0	$3.02x10^{-02}$	NS
DRB1*04:08	1 (0.3)	1 (0.5)	20 (10.3)	LaOT1D	1.76 [0.09-33.61]	NS	NS
				EOT1D	38.74 [6.96-405.1]	$1.28x10^{-08}$	**$7.03x10^{-07}$**
DQA1*03:01/ *03:02	50 (14.8)	67 (34.9)	67 (34.5)	LaOT1D	3.09 [2.04-4.74]	$2.08x10^{-07}$	**$4.98x10^{-06}$**
				EOT1D	3.04 [2.01-4.66]	$2.34x10^{-07}$	**$5.14x10^{-06}$**
DQA1*05:01	26 (7.7)	64 (33.3)	81 (41.8)	LaOT1D	6.00 [3.68-9.85]	$1.49x10^{-13}$	**$3.88x10^{-12}$**
				EOT1D	8.60 [5.24-13.92]	$1.68x10^{-20}$	**$4.54x10^{-19}$**
DQB1*02:01	22 (6.5)	70 (36.5)	90 (46.4)	LaOT1D	8.24 [4.89-13.78]	$8.74x10^{-18}$	**$3.06x10^{-16}$**
				EOT1D	12.43 [7.50-20.56]	$5.27x10^{-27}$	**$1.90x10^{-25}$**
DQB1*03:02	32 (9.5)	55 (28.6)	61 (31.4)	LaOT1D	3.84 [2.34-6.17]	$2.25x10^{-08}$	**$6.98x10^{-07}$**
				EOT1D	4.39 [2.72-6.97]	$4.89x10^{-10}$	**$1.61x10^{-08}$**

n (%): number and frequency of alleles in 169 control subjects, 96 LaOT1D subjects and 97 EOT1D subjects. Alleles shown have an allelic frequency ≥2.5% in patients (EOT1D and LaOT1D) or in controls. DQA1*03:01 and DQA1*03:02 alleles were pooled, as either with DQB1*02:01 constitute the DQ8 molecule. OR: Odds ratio; CI: Confidence interval; p-value, uncorrected; p-value (corrected), Holm-Bonferroni multi-comparison correction. Significant results are depicted in bold. NS, non-significant.

Table S4: HLA-DRB1, HLA-DQA1 and HLA-DQB1 protective alleles in EOT1D and LaOT1D.

Alleles	Controls n (%)	LaOT1D n (%)	EOT1D n (%)	Subject group	OR [95% CI]	p-value	p-value (corrected)
DRB1*07:01	53 (15.7)	18 (9.4)	9 (4.6)	LaOT1D	0.56 [0.31-0.98]	$4.64x10^{-02}$	NS
				EOT1D	0.26 [0.13-0.0.54]	$7.11x10^{-05}$	**$3.55x10^{-03}$**
DRB1*15:01	23 (6.8)	1 (0.5)	3 (1.5)	LaOT1D	0.07 [0.01-0.44]	$3.24x10^{-04}$	**$1.49x10^{-02}$**
				EOT1D	0.22 [0.07-0.69]	$5.95x10^{-03}$	NS
DQA1*01:02	60 (17.8)	9 (4.7)	10 (5.2)	LaOT1D	0.23 [0.11-0.0.46]	$6.80x10^{-06}$	**$1.43x10^{-04}$**
				EOT1D	0.25 [0.12-0.49]	$1.60x10^{-05}$	**$3.19x10^{-04}$**
DQA1*01:03	27 (8.0)	3 (1.6)	5 (2.6)	LaOT1D	0.18 [0.06-0.0.56]	$1.44x10^{-03}$	**$2.30x10^{-02}$**
				EOT1D	0.30 [0.13-0.78]	$1.28x10^{-02}$	NS
DQA1*02:01	53 (15.7)	18 (9.4)	9 (4.6)	LaOT1D	0.56 [0.31-0.98]	$4.64x10^{-02}$	NS
				EOT1D	0.26 [0.13-0.54]	$7.11x10^{-05}$	**$1.35x10^{-03}$**
DQA1*05:05	49 (14.5)	10 (5.2)	0 (0)	LaOT1D	0.32 [0.16-0.65]	$8.60x10^{-04}$	**$1.48x10^{-02}$**
				EOT1D	0	$6.95x10^{-11}$	**$1.74x10^{-09}$**
DQB1*02:02	50 (14.8)	24 (12.5)	0 (0)	LaOT1D	0.82 [0.49-1.36]	NS	NS
				EOT1D	0	$3.88x10^{-11}$	**$1.32x10^{-09}$**
DQB1*03:01	60 (17.8)	8 (4.2)	4 (2.1)	LaOT1D	0.20 [0.10-0.43]	$2.64x10^{-06}$	**$7.64x10^{-05}$**
				EOT1D	0.10 [0.04-0.26]	$4.44x10^{-09}$	**$1.42x10^{-07}$**
DQB1*06:02	21 (6.2)	1 (0.5)	1 (0.5)	LaOT1D	0.08 [0.01-0.43]	$9.79x10^{-04}$	**$2.74x10^{-02}$**
				EOT1D	0.08 [0.01-0.43]	$9.78x10^{-04}$	**$2.74x10^{-02}$**

n (%): number and frequency of alleles in 169 control subjects, 96 LaOT1D subjects and 97 EOT1D subjects. Alleles shown have an allelic frequency ≥2.5% in patients or in controls. OR: Odds ratio; CI: Confidence interval; p-value, uncorrected; p-value (corrected), Holm-Bonferroni multi-comparison correction. Significant results are depicted in bold. NS, non-significant.

Table S5: Susceptible and protective alleles defined by HLA-DQA1 position 52 in EOT1D and LaOT1D.

Haplotype DQA1 pos52	Controls n (%)	LaOT1D n (%)	EOT1D n (%)	Subject group	OR [95% CI]	p-value	p-value (corrected)
Risk alleles							
R	137 (40.5)	142 (74.0)	152 (78.4)	LaOT1D	4.17 [2.80-6.17]	7.01×10^{-14}	**7.01×10^{-13}**
				EOT1D	5.31 [3.54-8.00]	1.32×10^{-17}	**1.58×10^{-16}**
Protective alleles							
H	53 (15.7)	18 (9.4)	10 (5.2)	LaOT1D	0.56 [0.31-0.98]	4.64×10^{-02}	NS
				EOT1D	0.29 [0.14-0.58]	2.37×10^{-04}	**1.42×10^{-03}**
S	148 (43.8)	32 (16.7)	32 (16.5)	LaOT1D	0.26 [0.17-0.40]	1.00×10^{-10}	**7.02×10^{-10}**
				EOT1D	0.25 [0.16-0.39]	5.91×10^{-11}	**4.73×10^{-10}**
R^{neg}	201 (59.5)	50 (26.0)	42 (21.6)	LaOT1D	0.24 [0.16-0.36]	7.01×10^{-14}	**7.01×10^{-13}**
				EOT1D	0.19 [0.13-0.28]	1.32×10^{-17}	**1.58×10^{-16}**

n (%): number and frequency of alleles in 169 control subjects, 96 LaOT1D subjects and 97 EOT1D subjects. OR: Odds ratio; CI: Confidence interval; p-value, uncorrected; p-value (corrected), Holm-Bonferroni multi-comparison correction. Significant results are depicted in bold. NS, non significant.

Table S6: Susceptible and protective alleles defined by HLA-DQB1 position 57 in EOT1D and LaOT1D.

Haplotype DQB1 pos57	Controls n (%)	LaOT1D n (%)	EOT1D n (%)	Subject group	OR [95% CI]	p-value	p-value (corrected)
Risk alleles							
A	106 (31.4)	151 (78.6)	156 (80.4)	LaOT1D	8.06 [5.28-12.07]	$1.76x10^{-26}$	**$2.47x10^{-25}$**
				EOT1D	8.99 [5.93-10.72]	$1.19x10^{-28}$	**$1.78x10^{-27}$**
D^{neg}	193 (57.1)	178 (92.7)	182 (93.8)	LaOT1D	9.55 [5.28-17.61]	$5.99x10^{-20}$	**$6.59x10^{-19}$**
				EOT1D	11.39 [6.15-21.74]	$8.78x10^{-22}$	**$1.14x10^{-20}$**
Protective alleles							
D	145 (42.9)	14 (7.3)	12 (6.2)	LaOT1D	0.10 [0.06-0.19]	$5.99x10^{-20}$	**$6.59x10^{-19}$**
				EOT1D	0.09 [0.05-0.16]	$8.78x10^{-22}$	**$1.14x10^{-20}$**
V	69 (20.4)	23 (12.0)	19 (9.8)	LaOT1D	0.53 [0.32-0.87]	$1.67x10^{-02}$	NS
				EOT1D	0.42 [0.25-0.73]	$1.52x10^{-03}$	**$1.37x10^{-02}$**

n (%): number and frequency of alleles in 169 control subjects, 96 LaOT1D subjects and 97 EOT1D subjects. OR: Odds ratio; CI: Confidence interval; p-value, uncorrected; p-value (corrected), Holm-Bonferroni multi-comparison correction. Significant results are depicted in bold. NS, non significant.

Table S7: DR3 and DR4 susceptibility haplotypes in LaOT1D and EOT1D.

Haplotypes DRB1-DQA1-DQB1	Controls n (%)	LaOT1D n (%)	EOT1D n (%)	Subject group	OR [95% CI]	p-value	p-value (corrected)
03:01-05:01-02:01	22 (6.5)	60 (31.2)	78 (40.2)	LaOT1D	6.53 [3.81-11.04]	$1.83x10^{-13}$	**$9.69x10^{-12}$**
				EOT1D	9.66 [5.79-16.05]	$6.42x10^{-21}$	**$3.47x10^{-19}$**
04:01-03-03:02	4 (1.2)	17 (8.9)	18 (9.3)	LaOT1D	8.11 [2.71-22.46]	$2.51x10^{-05}$	**$1.21x10^{-03}$**
				EOT1D	8.54 [2.91-23.54]	$1.73x10^{-05}$	**$5.75x10^{-04}$**
04:02-03-03:02	8 (2.4)	11 (5.7)	15 (7.7)	LaOT1D	2.51 [1.02-6.13]	NS	NS
				EOT1D	3.46 [1.49-7.88]	$6.44x10^{-03}$	NS
04:04-03-03:02	5 (1.5)	8 (4.2)	9 (4.6)	LaOT1D	2.90 [0.91-7.94]	NS	NS
				EOT1D	3.24 [1.12-8.73]	$4.50x10^{-02}$	NS
04:05-03-03:02	7 (2.1)	16 (8.3)	0 (0)	LaOT1D	4.30 [1.81-11.40]	$1.30x10^{-03}$	NS
				EOT1D	0	NS	NS
04:08-03-03:02	0 (0)	1 (0.5)	17 (8.8)	LaOT1D	>1.76 [0.09-33.61]	NS	NS
				EOT1D	>32.37 [5.62-341.10]	$2.73x10^{-07}$	**$1.39x10^{-05}$**

n (%): number and frequency of haplotypes in 169 control subjects, 96 LaOT1D subjects and 97 EOT1D subjects. Haplotypes shown have a frequency ≥2.5% in patients or in controls. OR: Odds ratio; CI: Confidence interval; p-value, uncorrected; p-value (corrected), Holm-Bonferroni multi-comparison correction. Significant results are depicted in bold. NS, non-significant. For haplotypes absent in healthy controls, the value of n=1 was considered to calculate the OR.

Table S8: Protective DRB1-DQA1-DQB1 haplotypes in LaOT1D and EOT1D patients.

Haplotypes DRB1-DQA1-DQB1	Controls n (%)	LaOT1D n (%)	EOT1D n (%)	Patient group	OR [95% CI]	p-value	p-value (corrected)
01:01-01:01-05:01	17 (4.9)	7 (3.6)	9 (4.6)	LaOT1D	0.71 [0.27-1.66]	NS	NS
				EOT1D	0.92 [0.39-2.11]	NS	NS
01:02-01:01-05:01	20 (5.9)	7 (3.6)	6 (3.1)	LaOT1D	0.60 [0.24-1.43]	NS	NS
				EOT1D	0.51 [0.21-1.29]	NS	NS
07:01-02:01-02:01/ 02:02	48 (14.2)	14 (7.3)	8 (4.1)	LaOT1D	0.48 [0.26-0.87]	$1.71x10^{-02}$	NS
				EOT1D	0.26 [0.12-0.55]	$1.91x10^{-04}$	**$8.22x10^{-3}$**
08:01-04:01-04:02	9 (2.6)	0 (0)	2 (1.0)	LaOT1D	0	$2.99x10^{-02}$	NS
				EOT1D	0.38 [0.08-1.49]	NS	NS
11:01-05:05-03:01	21 (6.2)	4 (2.1)	0 (0)	LaOT1D	0.32 [0.12-0.90]	$3.31x10^{-02}$	NS
				EOT1D	0	$8.56x10^{-05}$	**$3.94x10^{-03}$**
13:01-01:03-06:03	23 (6.6)	3 (1.6)	3 (1.6)	LaOT1D	0.22 [0.07-0-69]	$6.07x10^{-03}$	NS
				EOT1D	0.22 [0.07-0.69]	$5.95x10^{-03}$	NS
13:02-01:02-06:04	13 (3.8)	2 (1.0)	3 (1.6)	LaOT1D	0.26 [0.06-1.01]	NS	NS
				EOT1D	0.39 [0.12-1.36]	NS	NS
13:03-05:05-03:01	12 (3.6)	1 (0.5)	0 (0)	LaOT1D	0.14 [0.01-0.91]	$3.79x10^{-02}$	NS
				EOT1D	0	$5.11x10^{-03}$	NS
14:01-01:01-05:03	13 (3.8)	0 (0)	0 (0)	LaOT1D	0	$5.69x10^{-03}$	NS
				EOT1D	0	$2.97x10^{-03}$	NS
15:01-01:02-06:02	20 (5.9)	0 (0)	1 (0.5)	LaOT1D	0	$1.60x10^{-04}$	**$7.03x10^{-03}$**
				EOT1D	0.08 [0.01-0.46]	$9.83x10^{-04}$	**$4.13x10^{-02}$**
16:01-01:02-05:02	14 (4.0)	3 (1.6)	6 (3.1)	LaOT1D	0.37 [0.11-1.23]	NS	NS
				EOT1D	0.74 [0.29-1.98]	NS	NS

n (%): number of alleles and frequency of alleles in 169 control subjects, 96 LaOT1D subjects and 97 EOT1D subjects. Haplotypes shown have a frequency ≥2.5% in patients or in controls. OR: Odds ratio; CI: Confidence interval; p-value, uncorrected; p-value (corrected), Holm-Bonferroni multi-comparison correction. Significant results are depicted in bold. NS, non significant.

Table S9: Risk and protective haplotypes defined by HLA-DQB1 position 57, HLA-DRB1 position 13 and HLA-DRB1 position 71 in EOT1D and LaOT1D.

Haplotype	Controls n (%)	LaOT1D n (%)	EOT1D n (%)	Subject group	OR [95% CI]	p-value	p-value (corrected)
Risk haplotypes							
A-H-K	5 (1.5)	20 (10.4)	22 (11.3)	LaOT1D	7.74 [3.04-19.13]	$5.97x10^{-06}$	**$4.59x10^{-04}$**
				EOT1D	8.52 [3.17-20.90]	$1.19x10^{-06}$	**$9.27x10^{-05}$**
A-S-K	21 (6.2)	62 (32.3)	78 (40.2)	LaOT1D	7.20 [4.17-12.41]	$8.91x10^{-15}$	**$7.13x10^{-13}$**
				EOT1D	10.15 [6.01-17.24]	$1.16x10^{-21}$	**$9.39x10^{-20}$**
Protective haplotypes							
D-G-R	18 (5.3)	0 (0)	3 (1.5)	LaOT1D	0	$3.00x10^{-04}$	**$2.22x10^{-02}$**
				EOT1D	0.28 [0.09-0.86]	$3.62x10^{-02}$	NS
D-R-A	24 (7.1)	0 (0)	3 (1.5)	LaOT1D	0	$2.91x10^{-05}$	**$2.21x10^{-03}$**
				EOT1D	0.21 [0.06-0.65]	$3.76x10^{-03}$	NS
D-S-E	29 (8.6)	5 (2.6)	3 (1.5)	LaOT1D	0.28 [0.12-0.71]	$5.65x10^{-03}$	NS
				EOT1D	0.17 [0.05-0.51]	$5.40x10^{-04}$	**$3.89x10^{-02}$**
D-S-R	41 (12.1)	6 (3.1)	1 (0.5)	LaOT1D	0.23 [0.10-0.54]	$3.61x10^{-04}$	**$2.67x10^{-02}$**
				EOT1D	0.04 [0-0.21]	$1.10x10^{-07}$	**$8.70x10^{-06}$**

n (%): number and frequency of haplotypes in 169 control subjects, 96 LaOT1D subjects and 97 EOT1D subjects. OR: Odds ratio; CI: Confidence interval; p-value, uncorrected; p-value (corrected), Holm-Bonferroni multi-comparison correction. Significant results are depicted in bold. NS, non significant.

Table S10: Genetic risk conferred by DR3 and DR4 haplotypes in LaOT1D and EOT1D, as determined by regularized binary logistic regression.

Haplotypes DRB1-DQA1-DQB1	Subject group	OR [97.5% CI]	p-value
03:01-05:01-02:01	LaOT1D	8.43 [6.83-10.73]	**<1x10^{-03}**
	EOT1D	11.00 [8.99-14.07]	**<1x10^{-03}**
04:01-03-03:02	LaOT1D	8.63 [6.17-12.29]	**<1x10^{-03}**
	EOT1D	8.35 [5.72-11.89]	**<1x10^{-03}**
04:02-03-03:02	LaOT1D	3.63 [2.45-5.34]	**<1x10^{-03}**
	EOT1D	6.12 [4.21-8.40]	**<1x10^{-03}**
04:04-03-03:02	LaOT1D	2.18 [1.26-2.86]	**6x10^{-03}**
	EOT1D	2.83 [2.00-3.80]	**<1x10^{-03}**
04:05-03-03:02	LaOT1D	6.46 [4.56-8.78]	**<1x10^{-03}**
	EOT1D	0.67 [0.61-0.78]	**<1x10^{-03}**
04:08-03-03:02	LaOT1D	1.81 [1.00-2.14]	**<1x10^{-03}**
	EOT1D	13.62 [9.26-17.38]	**<1x10^{-03}**

OR: Odds ratio, calculated using the formula: coefficient=ln(OR); CI: Confidence interval; p-values in binary logistic regression analysis are depicted. Significant results are depicted in bold.

Figure S1

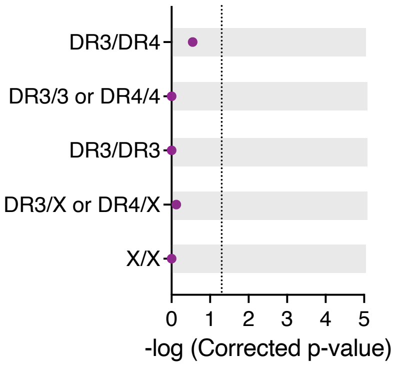

Allelic association tests in Early-onset T1D (EOT1D) versus Later onset T1D (LaOT1D) patients, represented as -log p-value, after Holm-Bonferroni correction. The dashed line represents -log p-value=0.05.

Figure S2

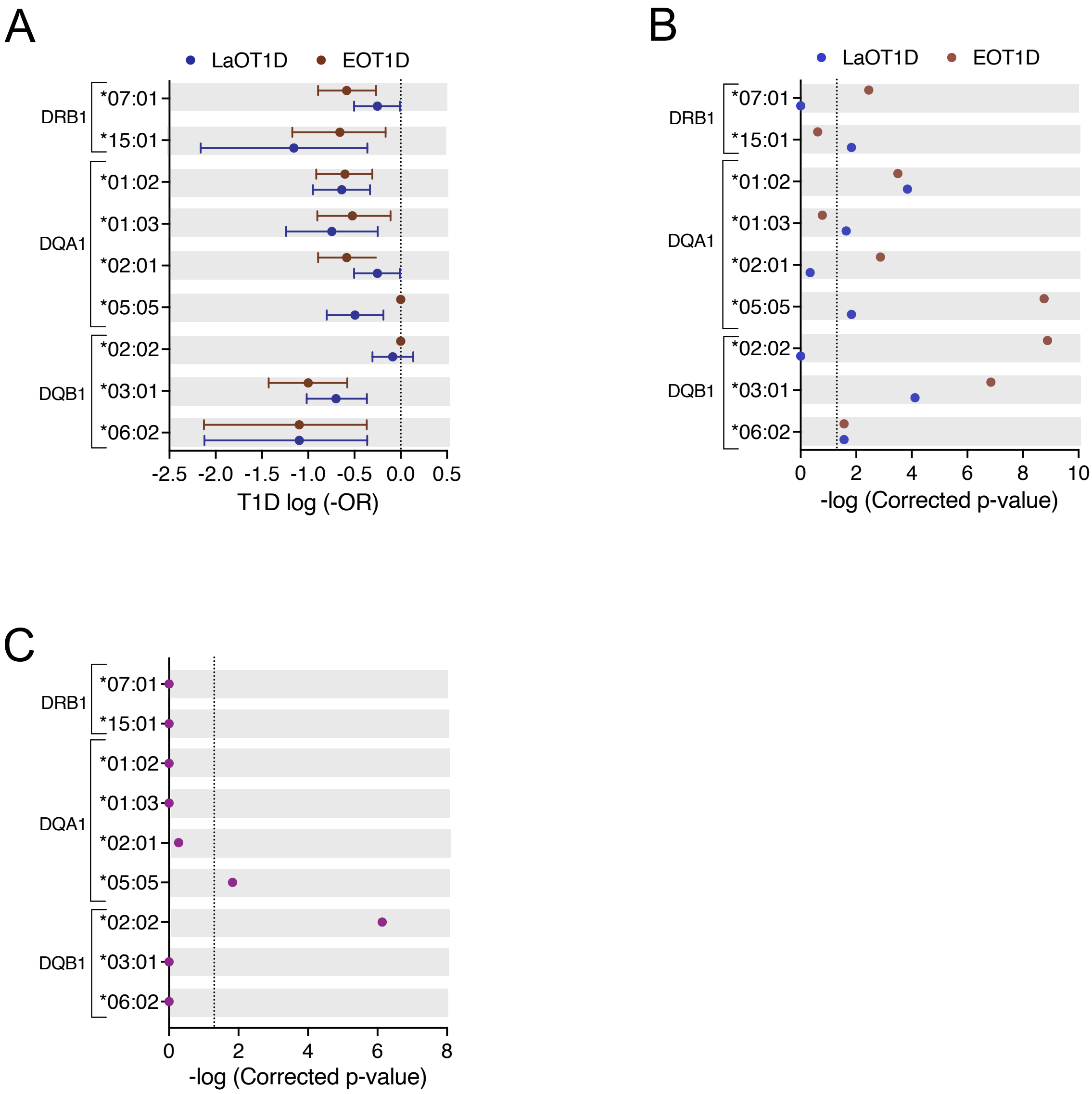

Genetic protection conferred by HLA class II alleles in EOT1D and LaOT1D patients. In A, the mean log -odds ratio (OR) ± 95% CI of HLA class II individual alleles in case versus controls is shown. The dashed line represents log -OR=0. B, allelic association tests in case versus controls, represented as -log p-value, after Holm-Bonferroni correction. EOT1D (n=97) versus control (n=169) comparisons are represented in red and LaOT1D (n=96) versus controls (n=169) in blue. In C, allelic association analyses in EOT1D versus LaOT1D patients are shown (purple). The dashed line in B and C represents -log p-value=0.05.

Figure S3

DRB1*04:01	1	GDTRPRFLEQVKHECHFFNGTERVRFLDRYFYHQEEYVRFDSDVGEYRAVTELGRP**D**AEY	60
DRB1*04:02	1	GDTRPRFLEQVKHECHFFNGTERVRFLDRYFYHQEEYVRFDSDVGEYRAVTELGRP**D**AEY	60
DRB1*04:04	1	GDTRPRFLEQVKHECHFFNGTERVRFLDRYFYHQEEYVRFDSDVGEYRAVTELGRP**D**AEY	60
DRB1*04:05	1	GDTRPRFLEQVKHECHFFNGTERVRFLDRYFYHQEEYVRFDSDVGEYRAVTELGRP**S**AEY	60
DRB1*04:08	1	GDTRPRFLEQVKHECHFFNGTERVRFLDRYFYHQEEYVRFDSDVGEYRAVTELGRP**D**AEY	60
DRB1*04:01	61	WNSQKD**L**LE**QK**RAAVDTYCRHNYGV**G**ESFTVQRRVYPEVTVYPAKTQPLQHHNLLVCSVN	120
DRB1*04:02	61	WNSQKD**I**LE**DE**RAAVDTYCRHNYGV**V**ESFTVQRRVYPEVTVYPAKTQPLQHHNLLVCSVN	120
DRB1*04:04	61	WNSQKD**L**LE**QR**RAAVDTYCRHNYGV**V**ESFTVQRRVYPEVTVYPAKTQPLQHHNLLVCSVN	120
DRB1*04:05	61	WNSQKD**L**LE**QR**RAAVDTYCRHNYGV**G**ESFTVQRRVYPEVTVYPAKTQPLQHHNLLVCSVN	120
DRB1*04:08	61	WNSQKD**L**LE**QR**RAAVDTYCRHNYGV**G**ESFTVQRRVYPEVTVYPAKTQPLQHHNLLVCSVN	120
DRB1*04:01	121	GFYPGSIEVRWFRNGQEEKTGVVSTGLIQNGDWTFQTLVMLETVPRSGEVYTCQVEHPSL	180
DRB1*04:02	121	GFYPGSIEVRWFRNGQEEKTGVVSTGLIQNGDWTFQTLVMLETVPRSGEVYTCQVEHPSL	180
DRB1*04:04	121	GFYPGSIEVRWFRNGQEEKTGVVSTGLIQNGDWTFQTLVMLETVPRSGEVYTCQVEHPSL	180
DRB1*04:05	121	GFYPGSIEVRWFRNGQEEKTGVVSTGLIQNGDWTFQTLVMLETVPRSGEVYTCQVEHPSL	180
DRB1*04:08	121	GFYPGSIEVRWFRNGQEEKTGVVSTGLIQNGDWTFQTLVMLETVPRSGEVYTCQVEHPSL	180
DRB1*04:01	181	TSPLTVEWRARSESAQSKMLSGVGGFVLGLLFLGAGLFIYFRNQKGHSGLQPTGFLS	237
DRB1*04:02	181	TSPLTVEWRARSESAQSKMLSGVGGFVLGLLFLGAGLFIYFRNQKGHSGLQPTGFLS	237
DRB1*04:04	181	TSPLTVEWRARSESAQSKMLSGVGGFVLGLLFLGAGLFIYFRNQKGHSGLQPTGFLS	237
DRB1*04:05	181	TSPLTVEWRARSESAQSKMLSGVGGFVLGLLFLGAGLFIYFRNQKGHSGLQPTGFLS	237
DRB1*04:08	181	TSPLTVEWRARSESAQSKMLSGVGGFVLGLLFLGAGLFIYFRNQKGHSGLQPTGFLS	237

Multiple sequence alignment of the indicated DRB1*04 alleles. Amino acid sequences of DRB1 alleles were obtained from the IMGT/HLA database (https://www.ebi.ac.uk/ipd/imgt/hla/alleles/). Polymorphic amino acid residues (Positions 57, 67, 70, 71 e 86) are shown in bold. Black and red label indicates neutral and susceptibility alleles found in this study, respectively.

Figure S4

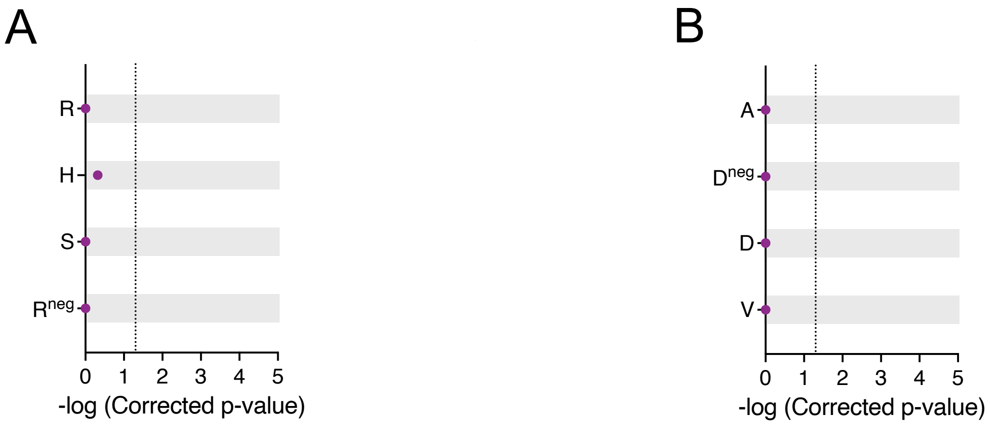

Allelic association analyses in EOT1D versus LaOT1D patients, represented as -log p-value, after Holm-Bonferroni correction. The dashed line indicates -log p-value=0.05. In A, risk and protective alleles defined by DQA1 amino acid position 52 are shown. In B, risk and protective alleles defined by DQB1 amino acid position 57 are depicted.

Figure S5

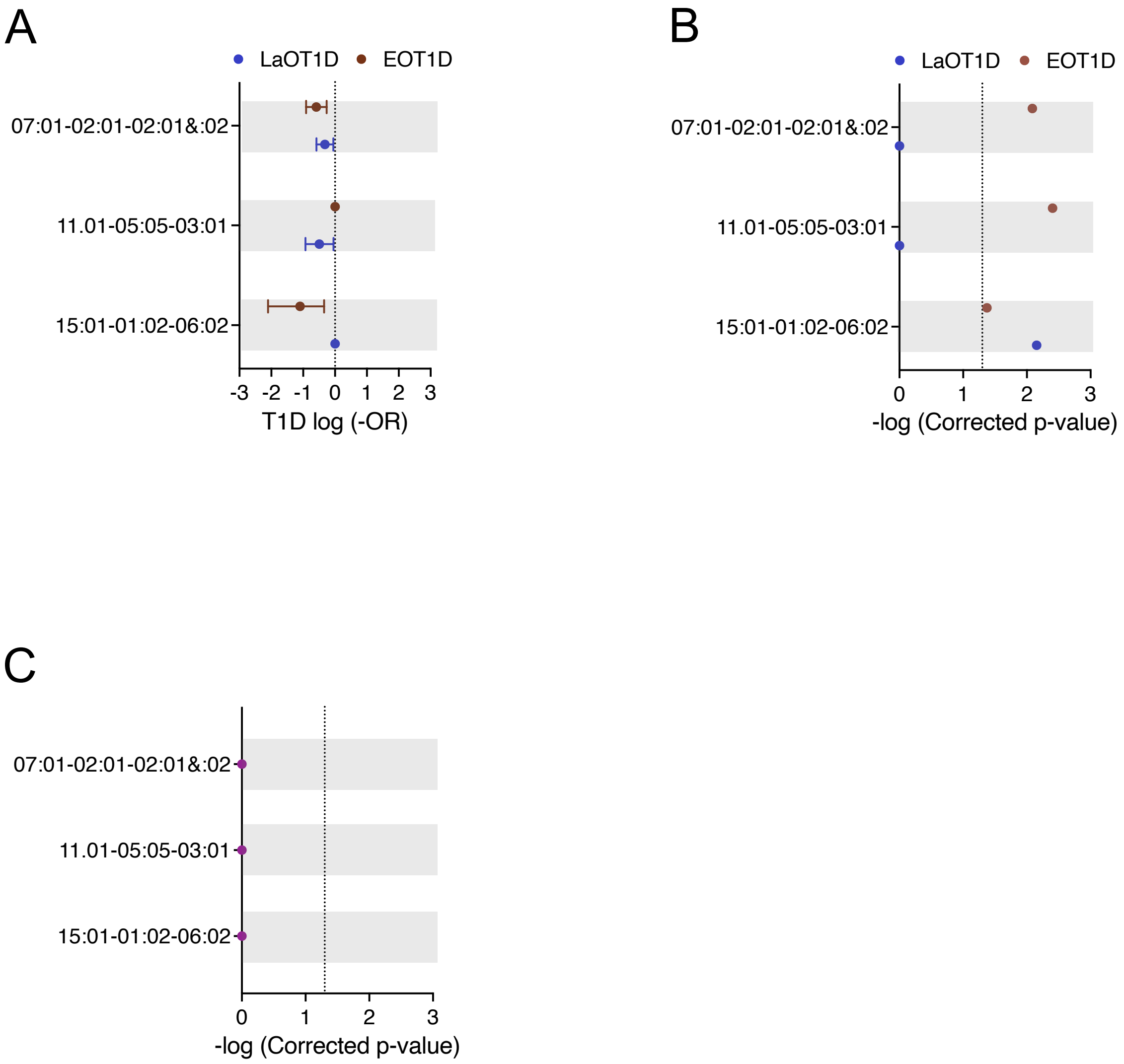

Genetic protection conferred by HLA class II haplotypes in EOT1D and LaOT1D patients. The mean log -odds ratio (OR) ± 95% CI of HLA class II individual alleles in case versus controls is presented in A. The dashed line represents log -OR=0. In B, allelic association tests in case versus controls, represented as -log p-value, after Holm-Bonferroni correction are depicted. EOT1D (n=97) versus control (n=169) comparisons are represented in red and LaOT1D (n=96) versus controls (n=169) in blue. In C, allelic association analyses in EOT1D versus LaOT1D patients are shown (purple). The dashed line in B and C represents -log p-value=0.05.

Figure S6

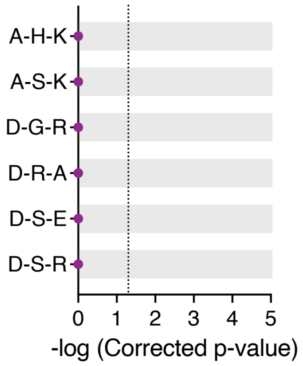

Allelic association analyses in EOT1D versus LaOT1D patients, represented as -log p-value, after Holm-Bonferroni correction. The dashed line indicates -log p-value=0.05. Plotted are the risk and protective haplotypes defined by DQB1 amino acid position 57 and DRB1 amino acid positions 13 and 71.

Figure S7

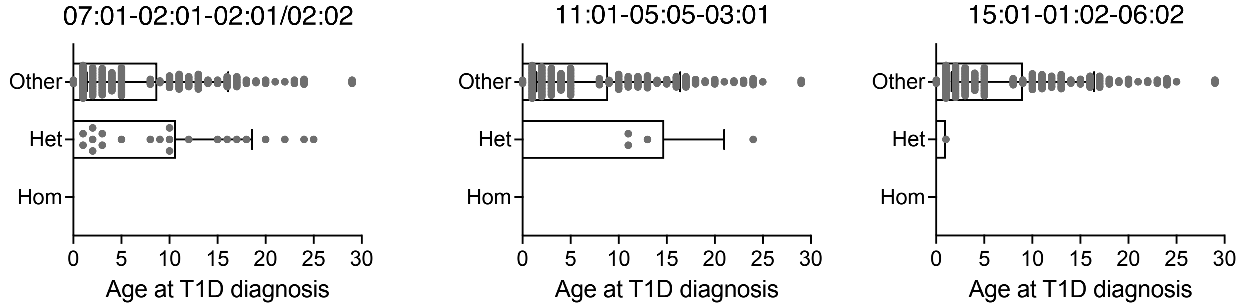

Genotypic stratification of age of T1D onset in patients (n=193) according to HLA class II haplotypes. Age distribution of homozygous and heterozygous subjects for the indicated protective haplotypes are plotted against all others.

Figure S8

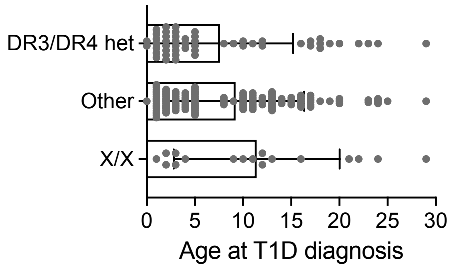

Genotypic stratification of age of T1D onset in patients (n=193) according to the indicated HLA class II genotypes. Age distribution of individuals heterozygous for DR3/DR4 are plotted against the age at T1D onset of individuals not harboring DR3 or DR4 haplotypes (X/X), and age at diagnosis of individuals homozygous or heterozygous for DR3 or DR4 (DR3/3, DR4/4, DR3/X and DR4/X; labeled as Other).
